# The effects of bariatric surgery on male and female fertility: A systematic review and meta-analysis

**DOI:** 10.1016/j.amsu.2022.103881

**Published:** 2022-06-15

**Authors:** Abdullah A. Al Qurashi, Syeda Hafsa Qadri, Sejal Lund, Ushna Sunain Ansari, Amna Arif, Amatul Rehman Durdana, Rabeata Maryam, Mahinn Saadi, Muhammad Zohaib, Muhammad Khuzzaim Khan, Areesha Waseem, Sophia Dar, Talal Almas

**Affiliations:** a College of Medicine, King Saud Bin Abdulaziz University for Health Sciences, Jeddah, Saudi Arabia; b King Abdullah International Medical Research Center, Jeddah, Saudi Arabia; c Dow University of Health Sciences, Karachi, Pakistan; d Shaheed Mohtarma Benazir Bhutto Medical University, Karachi, Pakistan; e United Medical and Dental College, Karachi, Pakistan; f Hackensack University Medical Center, Hackensack, NJ, USA; g Royal College of Surgeons in Ireland, Dublin, Ireland

**Keywords:** Bariatric surgery, Obesity, Sex hormones, Sexual function, Semen analysis

## Abstract

• Currently natural lifestyle modification is recommended over bariatric surgery in the management of infertility in obese men and women, despite natural weight loss often being an ineffective method to both lose and maintain weight loss in these individuals.
• Through this meta-analysis and systematic review, we provide evidence that bariatric surgery effectively improves fertility outcomes for men and women by measurements of hormone levels and improvements in sexual function index scores.
• This study demonstrates the importance and even cruciality of bariatric surgery in obese men and women who struggle with reproductive health, especially when finding it difficult to lose and maintain weight. It also proves that it is vital to continue to create and expand our knowledge with evidence-based medicine to help this cohort of patients.

## 1 Introduction

Obesity is a global epidemic estimated to affect 650 million adults worldwide with prevalence tripling over the recent decades [1]. Obesity negatively impacts the body's physiology and leads to various comorbidities, including infertility [2]. Irregular hormones and menstrual cycles are involved in the pathogenesis of infertility in obese women and poor sperm quality and hormonal disturbances contributes to infertility in obese men [3,4]. While nonsurgical weight reduction improves fertility parameters in both sexes, bariatric surgery is a more effective method in morbidly obese individuals [5,6]. Bariatric surgery improves fertility outcomes primarily through an improved hormonal profile [7]. The purpose of this study is to review the role of bariatric surgery on outcomes of fertility and reproductive function in men and women.

## 2 Methods

### 2.1 Patient and public involvement

The research question was designed to address a rising prevalence of a severe medical ailment-obesity and some dire consequences or associations these patients face with poor reproductive function. Patients were not directly involved in this meta-analysis and systemic review; however, all of the studies selected were either retrospective/prospective reviews or randomized control trials (RCTs) that directly interacted with patients or patient identifiable data. This study utilizes previously published and publicly available data and therefore also did not require an IRB. The results of the study will be presented upon publication to the general public.

### 2.2 Data sources and search strategy

This meta-analysis was carried out following the preferred reporting items for systematic review and meta-analyses (PRISMA) guidelines [8]. Moreover, all protocols enlisted in the AMSTAR 2 (21) checklist were followed. An electronic search of PubMed and Scopus was conducted from their inception to May 2021 without any language or publication year restrictions. Detailed search strategies are given in Supplementary Table 1. We manually screened the reference list of previous meta-analyses and reviewed articles to identify relevant studies.

### 2.3 Study selection

The following eligibility criteria were used: (a) RCTs or observational studies with a follow-up duration of 6 months and/or 12 months; (b) male and/or female participants undergoing bariatric surgery; (c) comparison between preoperative and post-op; (d) at least one fertility outcome reported from among the following: sexual function, sex hormones, semen analysis, menstrual irregularity. Studies that did not report total FSFI (Female Sexual Function Index) or erectile function component of IIEF (International Index of Erectile Dysfunction) scores were excluded. Studies reporting data as the median, interquartile range (IQR) were not included.

### 2.4 Data extraction and risk of bias assessment

Articles retrieved from the systematic search were exported to the EndNote Reference Library, version X20 (Clarivate Analytics), where duplicates were screened and removed. Two independent reviewers carefully evaluated the remaining articles, and studies that met defined criteria were selected. All trials were initially short-listed based on title and abstract and then subsequently reviewed to affirm relevance. A third investigator was consulted to resolve any discrepancies. Relevant authors were contacted via email in case of missing data. If the data were still not recovered, the corresponding studies were excluded. The following outcomes were extracted: total FSFI score, IIEF component of erectile function, male: total testosterone (TT), male: free testosterone (FT), male: estradiol (E2), male: follicle-stimulating hormone (FSH), male: luteinizing hormone (LH), male: sex hormone-binding globulin (SHBG), female: TT, female: FT, female: E2, female: FSH, female: LH, female: SHBG, total sperm count, semen volume, sperm motility, sperm morphology, sperm concentration, menstrual irregularity. The following baseline and study characteristics were extracted: first author, year of publication, country, study design, study population, type of surgery, sample size, mean age, mean preoperative BMI, mean postoperative BMI, and length of follow-up. The methodological index for non-randomized studies (MINORS) tool was used to assess the risk of bias [9].

### 2.5 Statistical analysis

RevMan (Version 5.4, The Cochrane Collaboration, 2020) was used for all statistical analyzes. The results of the studies were presented as mean difference (MD) or standard mean difference (SMD) for continuous variables and as risk ratios (RR) for dichotomous variables with 95% confidence intervals (CI). A *p-value* < 0.05 was considered statistically significant. All results were pooled using a random-effects model. Forest plots were created to assess visualize the results. Heterogeneity across studies was assessed using the I [2] statistic, with values of I [2] between 25% and 50% considered as mild heterogeneity, 50% and 75% regarded as moderate heterogeneity, and greater than 75% defined as severe heterogeneity [10]. Sensitivity analysis was performed for the following outcomes: male sex hormones, semen analysis, female sexual function (FSFI), and female sex hormones. The funnel plots were visually inspected for results with <10 studies to assess publication bias.

## 3 Results

### 3.1 Literature search results

**Fig. 1:**
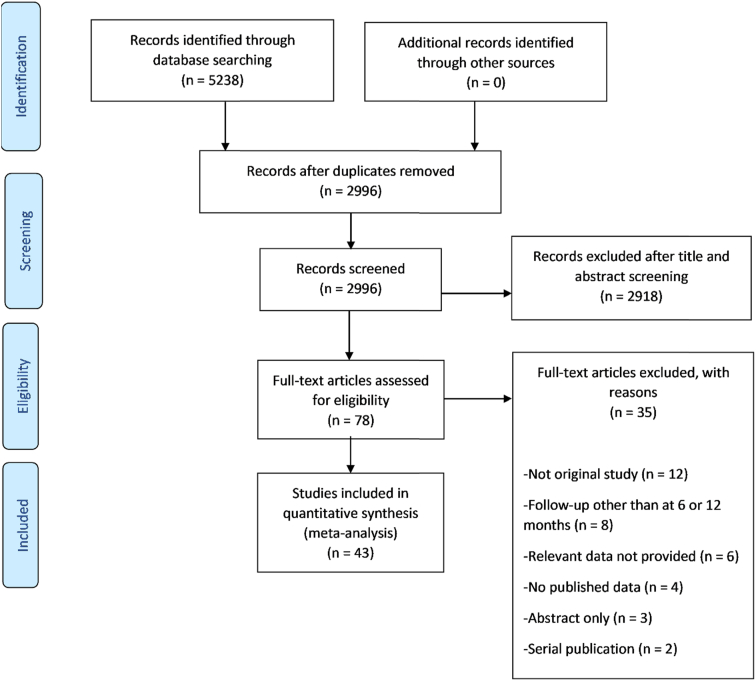
PRISMA flow diagram outlining literature search process.

The initial search yielded 5238 citations. After applying the predetermined inclusion/exclusion, 43 articles were ultimately included (38 prospective, three retrospectives, two not reported). The PRISMA flow chart (Fig. 1) summarizes the results of our literature search.

### 3.2 Study characteristics and risk of bias assessment

A total of 1,765 subjects are included in the analysis, out of which 753 are male and 1003 are female. Twenty-two studies reported male fertility outcomes, eighteen studies reported female fertility outcomes, and three studies reported both male and female fertility outcomes. The mean age of the individuals in the pooled sample at baseline is 36.88 ± 6.11 years (38.55 ± 5.83 years for males; 35.21 ± 6.40 years for females). The weighted mean BMI at baseline is 46.51 ± 6.28 kg/m^2^ (46.60 ± 6.42 kg/m^2^ for males; 46.42 ± 6.15 kg/m^2^ for females), which reduced to 26.70 ± 4.22 kg/m^2^ (30.14 ± 4.6 kg/m^2^ for males; 23.26 ± 3.84 kg/m^2^ for females) postoperatively at the 12-month follow-up. More than one type of bariatric surgery was performed in 18/43 studies (41.86%). Study characteristics and baseline demographics of the patients are summarized in Table 1. The mean MINORS score of included studies is 13 ± 1.25, ranging from eleven to sixteen, indicating moderate to high-quality evidence for non-randomized studies. Detailed assessment of each study is given in Supplementary Table 3. Visual assessment of funnel plots suggested that small studies appeared to be missing for male TT, FT, and SHBG (Supplementary Fig. 6).

**Table 1:** Patient characteristics.

Study/Author Name (Year)	Country	Study design	Study population	Surgery	N	Mean age (years)	Pre-BMI (mean ± SD) (kg/m2)	Post-BMI (mean ± SD) (kg/m2)	Follow-up (months)	Outcome indicators
Aarts [18] 2014	Netherlands	Prospective	M	LAGB, LRYGB	24	43.5 ± 2	46.1 ± 1.3	34.8 ± 0.8	12	1,2,3,4,5,6
Chin [19] 2018	New York	Retrospective cohort	M	GB	37	16.3 ± 2	48.2 ± 7.9	40.4 ± 6.8	12	1,4,5
Pellitero [20] 2012	Spain	Prospective	M	RYGB, SG	33	40.5 ± 9.9	50.3 ± 6.1	31.5 ± 4.7	12	1,2,3,6
Globerman [21] 2005	Israel	Prospective	M	VBG	17	38.2 ± 2.5	44.3 ± 1.7	31.6 ± 1.5	11.6 ± 1.4	1,2,4,5
Mora [22] 2013	Spain	Prospective	M	RYGB, SG	39	43.5 ± 10.5	46.9 ± 7.77	30.88 ± 5.04	12	1,2,3,4,5,6,8
Bastounis [23] 1998	Greece	Prospective	M + F	VBG	38 (F) 19 (M)	34.3 ± 5.9 (F)/34.7 ± 7.7 (M)	56.7 ± 7.7 (F)/57.1 ± 7.4 (M)	34.1 ± 4.8 (F)/34.7 ± 6.5 (M)	12	1,2,3,4,5,6 (F)/1,2,3,4,5,6 (M)
Mingrone [24] 2002	Italy	Prospective	M + F	BPD	31 (F) 15 (M)	30–45	48.3 ± 6.3 (F)/48 ± 5.4 (M)	35.2 ± 7.6 (F)/30.4 ± 3.5 (M)	12	6 (F)/6 (M)
Alagna [25] 2006	Italy	Prospective	M	BPD	20	21–63	47.3 ± 13.1	33.5 ± 7	12 ± 1	1,3,4,5
Woodard [26] 2012	USA	Prospective	M	RYGB	64	48.1 ± 1.3	48.2 ± 1.5	35.6 ± 1^a^/32.4 ± 1^b^	6, 12	1
Botella-Carretero [27] 2013	Spain	Prospective	M	BPD, RYGB, LAGB	20	40 ± 10.3	47.05 ± 5.99	35 ± 6.57	6	1,2,3,4,5,6
Ippersiel [28] 2013	Belgium	Prospective	M	RYGB, SG	21	40 (33–53)	45.3 ± 5.6	31 ± 4.2	12	1,2
Mihalca [29] 2014	Romania	Prospective	M	SG	28	43.07 ± 9.56	50.1 ± 11.19	35.87 ± 7.02	6	1,5,6
Samavat [30] 2014	Italy	Prospective	M	RYGB, LAGB,BPD, SG	55	42.3 ± 11.6	46.6 ± 7.4	37.5 ± 6.7^a^/32.2 ± 6.8^b^	6, 12	1,2,3,4,5,6
Legro [31] 2015	USA	Prospective cohort	M	RYGB	6	37.5 (30–40)	48 ± 7	35 ± 7a/32 ± 7b	6, 12	1,3,6,10,11,12,14
Sarwer [32] 2015	Pennsylvania	Prospective cohort	M	RYGB	32	48 (24–64)	45.1 (37.3–64.6)	NR	12	1,2,5,6,8
Kun [33] 2015	China	Retrospective cohort	M	RYGB	39	45.2 ± 12.3	41.2 ± 8.5	32.1 ± 7.3	12	1
Boonchaya-Anant [34] 2016	Thailand	Prospective	M	RYGB, SG	29	31 ± 8	56.9 ± 11.7	42.9 ± 9	6	1,2,3,6
Gao [35] 2018	China	Prospective	M	LSG	30	33 ± 9.5	40.2 ± 5.2	30.8 ± 4.4	6	1,2,3,4,5,6
Liu [36] 2018	China	Retrospective	M	RYGB	45	47 ± 9.97a/46.5 ± 9.71b	32.81 ± 4.04	25.48 ± 3.29^a^/25.41 ± 3.36^b^	6, 12	1,2
Samavat [37] 2018	Italy	Prospective	M	LRYGB	23	38 ± 9	45.8 ± 7.4	34.7 ± 5.3	6	1,2,3,4,5,6,10,11,12,13,14
Fariello [38] 2021	Brazil	Prospective	M	RYGB	15	20–50	45.7 ± 8.3	36.1 ± 6.4^a^/28.0 ± 2.8^b^	6, 12	1,2,3,4,5,6,10,11,12,13,14
Oncel [39] 2021	Turkey	Prospective	M	LSG	40	35.70 ± 4.22	47.20 ± 6.62	35.89 ± 4.95	6	1
Zhu [40] 2019	China	Prospective	M	LSG	56	30.8 ± 7.8	41.9 ± 5.8	26.1 ± 4.3	12	1,2
Ernst [41] 2013	Switzerland	Prospective	F	RYGB	36	41.2 ± 1.6	44.5 ± 0.8	27.9 ± 0.6	12	1,2,6
Legro [42] 2012	USA	Prospective cohort	F	RYGB	29	34.5 ± 4.3	49 ± 7	NR	6, 12	7
Sarwer [43] 2014	Pennsylvania	Prospective cohort	F	RYGB, LAGB	106	41 (34–48)	44.5 (41.4–49.7)	NR	12	1,3,4,5,6,7
Kjaer [44] 2017	Denmark	Prospective cohort	F	RYGB	31	34 (22–49)	44.1 ± 5.8	32.4 ± 9.8^a^/30.3 ± 5.8^b^	6, 12	1,2,3,4,5,6
Eid [45] 2014	Pennsylvania	Prospective	F	RYGB	14	36.3 ± 8.4	44.8 ± 1.6	32.4 ± 0^a^/29.2 ± 5.9^b^	6, 12	1,2,4,5,9
Escobar-Morreal [46] 2005	Spain	Prospective	F	BPD, LGB	17	29.8 ± 5.3	50.7 ± 7.1	NR	12 ± 5	1,2
Bhandari [47] 2016	India	Prospective	F	SG	75	28 ± 5	43.77 ± 5.9	31.71 ± 3.2	6	9
Turkmeen [48] 2015	Sweden	NR	F	LRYGB	8	31.4 ± 7.41	47.2 ± 8.85	35.7 ± 8.01^a^/32.82 ± 9.3^b^	6, 12	1,6,9
Dixon [49] 2011	Australia	NR	F	Lap-Band	42	34.0 ± 6.5	45.3 ± 7.3	36.4 ± 6.8	12	1,6
Carette [50] 2011	France	Prospective cohort	M	GB, SG	46	38.9 ± 7.9	44.1 ± 5.7	33.2 ± 5.4^a^/31.4 ± 5.3^b^	6, 12	12,13,14
Bond [51] 2011	USA	Prospective	F	RYGB, LAGB	54	43.3 ± 9.5	45.1 ± 6.8	NR	6	7
Whitcomb [52] 2012	USA	Prospective cohort	F	LGB, LSG	98	43.3 ± 11.8	39.7 ± 6.2	34.4a ±5.4^a^/34.0 ± 5.6^b^	6, 12	7
Hernández [53] 2013	Spain	Prospective	F	LBPD	80	43.5 ± 9.2	52.2 ± 8.2	NR	6, 12	7
Goitein [54] 2015	Israel	Prospective	F	LRYGB, SG	34	38.4 ± 9.1	44.4 ± 5.5	32.5 ± 5.1	6	7
Pichlerova [55] 2019	Czech Republic	Prospective	F	LAGB, BPD, Gastric Plication	60	41.7 ± 10.8	43.7 ± 5.99	36.4^a^	6, 12	7
Cherick [56] 2019	France	Prospective	F	SG, RYGB	36	37 ± 13	41 ± 7	29 ± 5	6	7
Lechmiannandan [57] 2019	Malaysia	Prospective	F	SG, GB	52	38.77 ± 6.7	39.89 ± 6.9	30.32 ± 5.4	6	7
Assimakopoulos [58] 2011	Greece	Prospective	F	BPD-LL, SG,/RYGB-LL	59	18–56	51.9 ± 9.92	31.8 ± 4.92	12	7
Efthymiou [59]/2015	Greece	Prospective	M + F	SG, RYGB, BPD	50	37.3 ± 9.6 (M)/37.2 ± 10.7 (F)	50.66 ± 7.9	NR	6, 12	7,8
Akan [60] 2018	Turkey	Prospective	F	LSG	53	34.85 ± 9.38	47.43 ± 6.37	37.77 ± 5.2	12	7

R = not reported, BMI = body mass index, F = Females, M = Males, n = number of participants.

RYGB = Roux-en-Y gastric bypass, LRYGB = laparoscopic Roux-en-Y gastric bypass, SG = sleeve gastrectomy, LSG = laparoscopic sleeve gastrectomy, LAGB = laparoscopic adjustable gastric band, LGB = laparoscopic gastric banding, BPD = biliopancreatic diversion, LBPD = laparoscopic biliopancreatic diversion, RYGB-LL = Roux-en-Y gastric bypass with long limb, BPD-LL = biliopancreatic diversion with Roux-en-Y reconstruction, GB = gastric bypass, VBG = Vertical banded gastroplasty, Lap-Band = laparoscopic banding BMI = body mass index, FSFI = Female Sexual Function Index, IIEF- erectile function = International Index of Erectile Function - component of IIEF, TT = total testosterone, FT = free testosterone, LH = luteinizing hormone, FSH = follicle stimulating hormone, SHBG = sex hormone–binding globulin, E2 = estradiol.

Outcome Indicators: 1 = (TT), 2 = (FT), 3 = (E2), 4 = (FSH), 5 = (LH), 6 = (SHBG), 7 = (FSFI), 8 = (IIEF- erectile function), 9 = Menstrual dysfunction, 10 = sperm concentration, 11 = sperm morphology, 12 = sperm motility, 13 = sperm count, 14 = semen volume, ^a^ = 6 months follow-up ^b^ = 12 months follow-up.

**Fig. 2:**
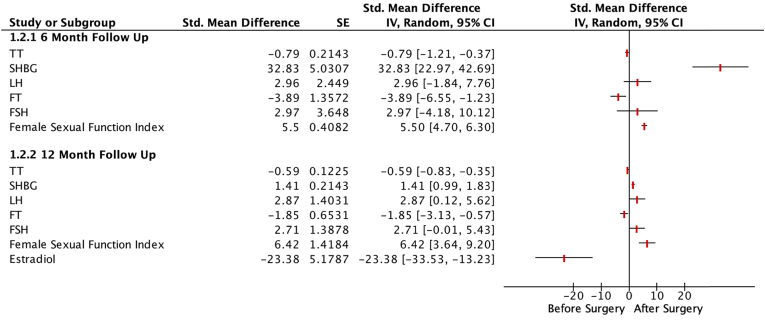
This Forrest plot summarizes the results of our meta-analysis demonstrating the effects of bariatric surgery on outcomes of fertility in females.

**Fig. 3:**
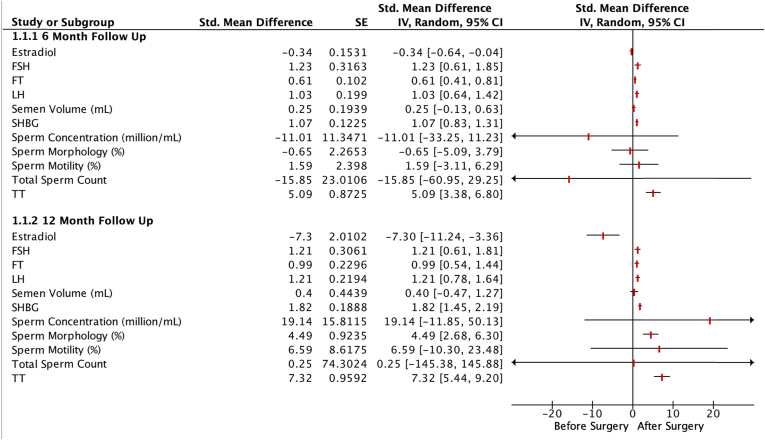
This Forrest plot summarizes the results of our meta-analysis demonstrating the effects of bariatric surgery on outcomes of fertility in males.

Outcome analysis: Fig. 2, Fig. 3 summarize the results of the study.

### 3.3 Male fertility

#### 3.3.1 Sexual function

Adequate data was provided for the component of erectile function of IIEF in 3 out of 43 studies in 101 patients. Bariatric surgery increased erectile function scores at 12 months follow-up (MD 4.01; 95% CI 1.63–6.39; P < 0.0010) (Supplementary Fig. 1).

#### 3.3.2 Sex hormones

From 43 studies included, 16 studies reported TT in 518 patients, 11 reported FT in 352 patients, 8 reported estradiol in 211 patients, 9 reported LH in 258 patients, 8 reported FSH in 226 patients and 10 reported SHBG in 294 patients. Bariatric surgery increased TT and FT levels at 12 months of follow-up (MD 7.32; 95% CI 5.44 to 9.20; P < 0.00001 and SMD 0.99; 95% CI 0.54 to 1.44; P < 0.0001 respectively), while leading to decreased estradiol levels (MD -7.30; 95% CI -11.24 to −3.36; P < 0.0003). LH, FSH, and SHBG also increased significantly at 12 months after bariatric surgery (MD 1.21; 95% CI 0.78 to 1.63; P < 0.00001 and MD 1.21; 95% CI 0.61 to 1.81; P < 0.0001 and SMD 1.82; 95% CI 1.45 to 2.18; P < 0.00001 respectively) (Supplementary Figs. 2a–f).

Sensitivity analysis for LH showed a decrease in heterogeneity from 52% to 19% after removing the study by Mora et al. [11] (MD 1.37; 95% CI 1.05 to 1.68; P < 0.00001). Removal of study by Chin et al. [12] from the FSH results caused the heterogeneity to decrease from 51% to 0% (MD 1.37; 95% CI 0.98 to 1.77; P < 0.00001) for FSH. Sensitivity analysis for other sex hormones did not show any significant change.

#### 3.3.3 Semen analysis

Three studies reported semen volume and sperm motility in 65 patients; two reported total sperm count in 61 patients, and two reported sperm morphology and concentration in 19 patients at 12 months follow-up. Only sperm morphology increased 12 months after bariatric surgery (MD 4.49; 95% CI 2.68 to 6.29; P = 0.00001), while the other semen parameters, including total sperm count, semen volume, sperm motility and concentration did not significantly change (MD 0.25; 95% CI -145.38 to 145.88; P = 1 and MD 0.40; 95% CI -0.47 to 1.26; P = 0.37 and MD 6.59; 95% CI -10.30 to 23.49; P = 0.44 and MD 19.14; 95% CI -11.85 to 50.14; P = 0.23, respectively) (Supplementary Figs. 3a–e).

For semen volume and sperm motility, removal of the Carette et al. resulted in a decrease in heterogeneity from 67% to 89%, respectively, to 0% for both outcomes [13]. Semen volume and sperm motility also became statistically significant (MD 0.93; 95% CI 0.29 to 1.57; P = 0.04 and MD 15.87; 95% CI 8.62 to 23.12; P < 0.0001 respectively).

### 3.4 Female fertility

#### 3.4.1 Sexual function

8 out of 43 selected studies reported total FSFI scores in 535 patients. The FSFI scores increased at the 12 months follow-up after bariatric surgery (MD 6.42; 95% CI 3.64 to 9.20; P < 0.00001) (Supplementary Fig. 4).

Sensitivity analysis showed a decrease in the heterogeneity of 83%–44% after removing the study by Hernàndez et al. [52] (MD 5.61; 95% CI 3.54 to 7.68; P < 0.00001) [14].

#### 3.4.2 Sex hormones

Of the 43 included studies, 7 studies reported TT in 275 patients, 4 reported FT in 119 patients, 3 reported estradiol in 175 patients, 4 reported LH and FSH in 189 patients, and 7 reported SHBG in 292 patients. Bariatric surgery significantly increased LH, FSH, and SHBG at the 12 months follow-up (MD 2.87; 95% CI 0.12 to 5.61; P = 0.04 and MD 2.71; 95% CI -0.01 to 5.43; P = 0.05 and SMD 1.41, 95% CI 0.99 to 1.83; P < 0.00001 respectively). TT, FT, and estradiol levels decreased (MD -0.59; 95% CI -0.83 to −0.36; P < 0.00001 and SMD -1.85; 95% CI -3.13 to −0.58; P = 0.004 and MD -23.38; 95% CI -33.53 to −13.23; P < 0.00001 respectively) (Supplementary Figs. 5a–f). Sensitivity analysis did not significantly impact heterogeneity.

#### 3.4.3 Menstrual irregularity

2 out of 43 studies reported menstrual irregularities in 23 patients. Bariatric surgery did not significantly affect menstrual irregularity at the 12 months follow-up (RR 0.20; 95% CI 0.01 to 7.17; P = 0.38) (Supplementary Fig. 6).

Results for 12- and 6-month follow-up are given in supplementary figures 1-6 and 6-12, respectively.

## 4 Discussion

We evaluated the effects of bariatric surgery on fertility in both males and females by analyzing changes in sex hormone levels, sperm parameters, menstrual irregularities, and sexual function.

### 4.1 Effects of bariatric surgery on male fertility

Our meta-analysis showed that one year after surgery, the levels of TT, FT, FSH, LH, and SHBG increased significantly in males, while estradiol showed a significant decrease. Along with improvements in sex hormones, our analysis showed a substantial increase in the erectile function domain of IIEF at 12-month follow-up. Unlike sex hormones and erectile function, sperm parameters, i.e., total sperm count, sperm motility, semen volume, and sperm concentration, except sperm morphology, did not significant change.

Multiple factors improve sex hormones in massively obese men post-bariatric surgery. Adipose tissue is a significant source of estrogen production in men and women as it contains the enzyme aromatase cytochrome P450 converting testosterone to estradiol (E2) [15]. Increased estradiol production can also inhibit the secretion of LH and FSH from the pituitary, which can decrease testosterone (both free and total) synthesis and spermatogenesis and ultimately lead to infertility. Low levels of SHBG in obesity decrease testosterone levels. Hyperinsulinemia or the low-grade inflammation and changes in proinflammatory/anti-inflammatory cytokines (i.e., TNF α, IL-1 β, and adiponectin) are also derived from adipose tissue [16]. Our study shows that the substantial loss of adiposity after bariatric surgery reduces aromatase activity, improves insulin sensitivity, regulates sex hormone levels, decreases E2 production, and increases testosterone hormone levels, SHBG FSH, and LH in the body [17]. This automatically regulates spermatogenesis. Bariatric surgery also regulates androgen and proinflammatory cytokine levels and, therefore, reduces erectile dysfunction [7]. According to our analysis, bariatric surgery did not significantly affect semen analysis parameters. The observational and controlled prospective study by Carette et al. (N = 26) reported worsened sperm parameters at postoperative follow-up of 1-year; which can be attributed to the nutritional deficiencies commonly seen after bariatric surgery [13]. Nutritional deficiency of vitamins and minerals, including iron and calcium, are crucial for spermatogenesis, and therefore the lack thereof may lead to the insignificant improvement on sperm quality parameters. In our analysis, the prospective study, Fariello et al. (N = 15) did not show significant improvement in the 12th postoperative month in all sperm parameters [18]. Whether bariatric surgery improves sperm parameters is controversial and requires further research.

### 4.2 Effects of bariatric surgery on female fertility

Unlike in obese males, obese females had a significant decrease in TT and FT after bariatric surgery. Bariatric surgery also led to lower E2 levels and increased LH, FSH, and SHBG levels. Sexual function reflected by FSFI scores also improved. A previous meta-analysis by Wen et al. did not show a statistically significant improvement in FSFI [19]. Although we found a statistically significant decrease in menstrual irregularities in the 6th postoperative month, we did not observe any statistically significant change at the 12-month follow up.

Although obesity leads to androgen deficiency in men, it causes excessive androgen production in women. The reduction of testosterone post-bariatric surgery can be due to increased synthesis of SHBG, which is otherwise inhibited by obesity-related insulin resistance and hyperinsulinemia [20]. The improved insulin sensitivity also helps in decreasing ovarian androgen production [16]. With extensive weight loss, the aromatization of testosterone decreases leading to better regulation of the FSH/LH ratios after surgery. Studies on whether sexual dysfunction in women improves after surgery are inconsistent thus far. These studies find that obese women with anovulatory cycles increased their chances of conception with bariatric surgery; improved insulin sensitivity with a significant drop in insulin levels after surgery helps regulate menstrual cycles, and therefore directly affects the chances of conception [16]. As mentioned above, we found a statistically significant improvement in menstrual irregularities at 6-month follow-up; however, the lack of significant changes observed at 12-month follow-up can be explained by a small pooled sample size since only 2 studies were available to review. The results of this meta-analysis highlight the integral correlation between bariatric surgery and the reproductive hormone levels and sexual function in men and women. Future research is required to further strengthen the evidence and highlight the role of underlying comorbidities e.g., diabetes, and hypertension on the outcomes of fertility in men and women.

### 4.3 Limitations

Our meta-analysis has several limitations. First, there was significant heterogeneity in the analysis of all results except the IIEF erectile function domain, sperm morphology, sperm concentration, and estradiol in women. This may be attributed to the absence of RCTs, different types of bariatric surgery, variable patient populations, and small population sizes of the included studies. Second, we did not evaluate sperm DNA fragmentation; only semen analysis was assessed to analyze any changes in the quality of sperm post-surgery. Third, although we improved on previous studies by analyzing all data at a specific follow-up time to reduce heterogeneity, this meta-analysis showed effects of bariatric surgery only up to one year. Long-term complications were not evaluated. Fourth, investigation of the influence of bariatric surgery on sexual function by self-reported questionnaires may be subject to response bias. Fifth, sexual function may also be affected by the quality of life, body image, and psychological state associated with weight loss; however, we did not analyze these factors. Finally, we did not evaluate fertility as a separate outcome to determine the incidence of successful pregnancies after surgery in infertile individuals due to the lack of studies that meet our eligibility criteria.

### 4.4 Conclusion

The results of our meta-analysis showed bariatric surgery significantly improves reproductive hormone levels and sexual function in men and women. Larger RCTs with longer follow-up times are essential to confirm our conclusions. Inconsistencies in the results of menstrual irregularities at six and twelve-month follow-up, inconclusive results of sperm analysis, and heterogeneity in several outcome analyses require higher-quality studies in the future to effectively conclude the role of bariatric surgery on outcomes of fertility in men and women.

## Ethical approval

As this study uses publicly available data published online, this study did not need IRB approval.

## Sources of funding for your research

There is no funding for this research.

## Author contribution

Abdullah Qureishi – contributed to study design and concept, writing the paper, and finalizing the paper prior to pubmission Syeda Hafsa Qadri contributed to study design, data collection, writing the paper Sejal Lund contributed to data collection, data analysis and writing the paper Ushna Sunain Ansari contributed to data collection, data analysis and writing the paper Amna Arif contributed to data collection and writing the paper Amatul Rehman Durdana contributed to data collection and writing the paper Rabeata Maryam contributed to data collection and writing the paper Mahinn Saadi contributed to writing the paper Muhammad Zohaib contributed to writing the paper Muhammad Khuzzaim Khan contributed to writing the paper Areesha Waseem contributed to writing the paper Sophia Dar contributed to writing the paper, editing and finalizing the paper Talal Almas contributed to writing the paper, editing and finalizing the paper.

## Data availability statement

Publicly available data was used available on pubmed.gov.

## Registration of research studies

1. Name of the registry:
2. Unique Identifying number or registration ID:
3. Hyperlink to your specific registration (must be publicly accessible and will be checked):

## Guarantor

Sophia Dar and Talal Almas accept full responsibility for the work and the conduction of the study.

## Permission to reproduce material from other sources

Not needed because publicly available data was used.

## Provenance

Not commissioned.

## Peer review

Externally peer-reviewed.

## Consent

This study did not directly involve patient care and has no patient identifiers.

## Declaration of competing interest

None of the authors have any conflicts of interest in this study.
