# Supplementary material for: The effects of bariatric surgery on male and female fertility: A systematic review and meta-analysis
Source: Ann Med Surg (Lond). 2022 Jun 15;80:103881. doi: 10.1016/j.amsu.2022.103881 (PMC9422049; doi:10.1016/j.amsu.2022.103881)
Supplement: Multimedia component 2 [file mmc2.docx]

**SUPPLEMENTAL APPENDIX**

**Table S1.** Search strategy used in each database searched

**Table S2.** Baseline Characteristics of Included studies

**Table s3.** Risk of bias assessment: MINORS criterion

**Figure S1.** PRISMA Flow diagram for retrieval of articles

**Figure S2.** Forest plot for erectile function component of IIEF at 12-month follow-up

**Figure S3.** Forest plots for male sex hormones at 12-month follow-up (a) male TT (nM), (b) male FT (nM), (c) male estradiol (pg/mL), (d) male LH (mIU/mL), (e) male FSH (mIU/mL), (f) male SHBG (nM)

**Figure S4.** Forest plots for semen analysis at 12 months of follow-up (a) total sperm count (10^6^), (b) semen volume (mL), (c) sperm motility (%), (d) sperm morphology (%), (e) sperm concentration (million / mL)

**Figure S5.** Forest plot of the total FSFI score at 12 months of follow-up

**Figure S6.** Forest plots for female sex hormones at 12-month follow-up (a) female TT (nM), (b) female FT (nM), (c) female estradiol (pg/mL), (d) female LH (mIU/mL), (e) female FSH (mIU/mL), (f) female SHBG (nM)

**Figure S7.** Forest plot for menstrual irregularity at 12-month follow-up

**Figure S8.** Forest plots for male sex hormones at 6-month follow-up (a) male TT (nM), (b) male FT (nM), (c) male estradiol (pg/mL), (d) male LH (mIU/mL), (e) male FSH (mIU/mL), (f) male SHBG (nM)

**Figure S9.** Forest plots for semen analysis at 6 months of follow-up (a) total sperm count (10^6^), (b) semen volume (mL), (c) sperm motility (%), (d) sperm morphology (%), (e) sperm concentration (million/mL)

**Figure S10.** Forest plot for the total FSFI score at 6 months of follow-up

**Figure S11.** Forest plots for female sex hormones at 6-month follow-up (a) female TT (nM), (b) female FT (nM), (c) female LH (mIU/mL), (d) female FSH (mIU/mL), (e) female SHBG (nM)

**Figure S12.** Forest plot of menstrual irregularity at 6 months of follow-up

**Figure S13.** Funnel plots for male sex hormones at 12-month follow-up (a) TT, (b) FT, (c) SHB

| **Table S1.** Search strategy used in each database searched | |
| --- | --- |
| **Database** | **Search strategy** |
| PubMed (articles retrieved= 2998) | ("bariatric surgery"[MeSH Terms] OR ("bariatric"[All Fields] AND "surgery"[All Fields]) OR "bariatric surgery"[All Fields] OR ("bariatric"[All Fields] OR "bariatrics"[MeSH Terms] OR "bariatrics"[All Fields]) OR ("bariatric surgery"[MeSH Terms] OR ("bariatric"[All Fields] AND "surgery"[All Fields]) OR "bariatric surgery"[All Fields] OR ("bariatric"[All Fields] AND "surgical"[All Fields] AND "procedures"[All Fields]) OR "bariatric surgical procedures"[All Fields]) OR ("gastric bypass"[MeSH Terms] OR ("gastric"[All Fields] AND "bypass"[All Fields]) OR "gastric bypass"[All Fields] OR "roux en gastric bypass"[All Fields]) OR (("sleeve"[All Fields] OR "sleeved"[All Fields] OR "sleeves"[All Fields] OR "sleeving"[All Fields]) AND ("gastrectomy"[MeSH Terms] OR "gastrectomy"[All Fields] OR "gastrectomies"[All Fields])) OR (("duodenitis"[MeSH Terms] OR "duodenitis"[All Fields] OR "duodenum"[MeSH Terms] OR "duodenum"[All Fields] OR "duodenal"[All Fields]) AND ("jejunally"[All Fields] OR "jejune"[All Fields] OR "jejunitis"[All Fields] OR "jejunum"[MeSH Terms] OR "jejunum"[All Fields] OR "jejunal"[All Fields]) AND ("bypass"[All Fields] OR "bypassed"[All Fields] OR "bypasses"[All Fields] OR "bypassing"[All Fields]) AND ("sleeve"[All Fields] OR "sleeved"[All Fields] OR "sleeves"[All Fields] OR "sleeving"[All Fields]) AND ("gastrectomy"[MeSH Terms] OR "gastrectomy"[All Fields] OR "gastrectomies"[All Fields])) OR (("duodenitis"[MeSH Terms] OR "duodenitis"[All Fields] OR "duodenum"[MeSH Terms] OR "duodenum"[All Fields] OR "duodenal"[All Fields]) AND ("switch"[All Fields] OR "switched"[All Fields] OR "switches"[All Fields] OR "switching"[All Fields] OR "switchings"[All Fields])) OR ("Revisional"[All Fields] AND ("surgery"[MeSH Subheading] OR "surgery"[All Fields] OR "surgical procedures, operative"[MeSH Terms] OR ("surgical"[All Fields] AND "procedures"[All Fields] AND "operative"[All Fields]) OR "operative surgical procedures"[All Fields] OR "general surgery"[MeSH Terms] OR ("general"[All Fields] AND "surgery"[All Fields]) OR "general surgery"[All Fields] OR "surgery s"[All Fields] OR "surgerys"[All Fields] OR "surgeries"[All Fields])) OR (("laparoscopes"[MeSH Terms] OR "laparoscopes"[All Fields] OR "laparoscope"[All Fields] OR "laparoscopical"[All Fields] OR "laparoscopically"[All Fields] OR "laparoscopics"[All Fields] OR "laparoscopy"[MeSH Terms] OR "laparoscopy"[All Fields] OR "laparoscopic"[All Fields]) AND ("adjustability"[All Fields] OR "adjustable"[All Fields] OR "adjustables"[All Fields] OR "adjustible"[All Fields]) AND ("gastrics"[All Fields] OR "stomach"[MeSH Terms] OR "stomach"[All Fields] OR "gastric"[All Fields]) AND ("banded"[All Fields] OR "banding"[All Fields] OR "bandings"[All Fields]))) AND ("conceptional"[All Fields] OR "conceptive"[All Fields] OR "fertilization"[MeSH Terms] OR "fertilization"[All Fields] OR "conception"[All Fields] OR "conceptions"[All Fields] OR (("hormon"[All Fields] OR "hormonal"[All Fields] OR "hormonally"[All Fields] OR "hormonals"[All Fields] OR "hormone s"[All Fields] OR "hormones"[Pharmacological Action] OR "hormones"[MeSH Terms] OR "hormones"[All Fields] OR "hormone"[All Fields] OR "hormons"[All Fields]) AND ("level"[All Fields] OR "levels"[All Fields])) OR ("gonadal steroid hormones"[MeSH Terms] OR ("gonadal"[All Fields] AND "steroid"[All Fields] AND "hormones"[All Fields]) OR "gonadal steroid hormones"[All Fields] OR ("sex"[All Fields] AND "hormones"[All Fields]) OR "sex hormones"[All Fields]) OR (("sperm s"[All Fields] OR "spermatozoa"[MeSH Terms] OR "spermatozoa"[All Fields] OR "sperm"[All Fields] OR "sperms"[All Fields]) AND ("qualities"[All Fields] OR "quality"[All Fields] OR "quality s"[All Fields])) OR (("sperm s"[All Fields] OR "spermatozoa"[MeSH Terms] OR "spermatozoa"[All Fields] OR "sperm"[All Fields] OR "sperms"[All Fields]) AND ("quantities"[All Fields] OR "quantity"[All Fields])) OR ("pregnancy"[MeSH Terms] OR "pregnancy"[All Fields] OR "pregnancies"[All Fields] OR "pregnancy s"[All Fields]) OR ("fertiles"[All Fields] OR "fertility"[MeSH Terms] OR "fertility"[All Fields] OR "fertile"[All Fields] OR "fertilities"[All Fields]) OR ("infertiles"[All Fields] OR "infertilities"[All Fields] OR "infertility"[MeSH Terms] OR "infertility"[All Fields] OR "infertile"[All Fields] OR "infertility s"[All Fields]) OR ("infertility"[MeSH Terms] OR "infertility"[All Fields] OR "subfertility"[All Fields] OR "subfertile"[All Fields]) OR (("sexual behavior"[MeSH Terms] OR ("sexual"[All Fields] AND "behavior"[All Fields]) OR "sexual behavior"[All Fields] OR "sexual"[All Fields] OR "sexually"[All Fields] OR "sexualities"[All Fields] OR "sexuality"[MeSH Terms] OR "sexuality"[All Fields] OR "sexualization"[All Fields] OR "sexualize"[All Fields] OR "sexualized"[All Fields] OR "sexualizing"[All Fields] OR "sexuals"[All Fields]) AND ("functional"[All Fields] OR "functional s"[All Fields] OR "functionalities"[All Fields] OR "functionality"[All Fields] OR "functionalization"[All Fields] OR "functionalizations"[All Fields] OR "functionalize"[All Fields] OR "functionalized"[All Fields] OR "functionalizes"[All Fields] OR "functionalizing"[All Fields] OR "functionally"[All Fields] OR "functionals"[All Fields] OR "functioned"[All Fields] OR "functioning"[All Fields] OR "functionings"[All Fields] OR "functions"[All Fields] OR "physiology"[MeSH Subheading] OR "physiology"[All Fields] OR "function"[All Fields] OR "physiology"[MeSH Terms])) OR ("reproductive health"[MeSH Terms] OR ("reproductive"[All Fields] AND "health"[All Fields]) OR "reproductive health"[All Fields])) |
| Scopus (articles retrieved= 2240) | ( ( TITLE-ABS-KEY ( bariatric AND surgery ) OR TITLE-ABS-KEY ( bariatrics ) OR TITLE-ABS-KEY ( bariatric AND surgical AND procedures ) OR TITLE-ABS-KEY ( Roux-en-Y AND Gastric AND Bypass ) OR TITLE-ABS-KEY ( Sleeve AND Gastrectomy ) OR TITLE-ABS-KEY ( Duodenal AND Jejunal AND Bypass AND with AND Sleeve AND Gastrectomy ) OR TITLE-ABS-KEY ( Duodenal AND Switch ) OR TITLE-ABS-KEY ( Revisional AND Surgery ) OR TITLE-ABS-KEY ( Laparoscopic AND Adjustable AND Gastric AND Banding ) ) ) AND ( ( TITLE-ABS-KEY ( conception ) OR TITLE-ABSKEY ( hormone AND levels ) OR TITLE-ABSKEY ( sex AND hormones) OR TITLE-ABS-KEY ( sex AND hormones ) OR TITLE-ABS-KEY ( sperm AND quality ) OR TITLE-ABS-KEY ( sperm AND quantity ) OR TITLE-ABS-KEY ( pregnancy ) OR TITLE-ABS-KEY ( fertility ) OR TITLE-ABSKEY ( infertility ) OR TITLE-ABS-KEY ( subfertility ) OR TITLE-ABS-KEY ( sexual AND function ) OR TITLE-ABS-KEY ( reproductive AND health ) ) ) |

| **Table S2.** Baseline Characteristics of Included studies | | | | | | | | | | |
| --- | --- | --- | --- | --- | --- | --- | --- | --- | --- | --- |
| **Study/Author Name (Year)** | **Country** | **Study design** | **Study population** | **Surgery** | **N** | **Mean age (years)** | **Pre-BMI (mean ± SD) (kg/m2)** | **Post-BMI (mean ± SD) (kg/m2)** | **Follow-up (months)** | **Outcome indicators** |
| Aarts [18] 2014 | Netherlands | Prospective | M | LAGB, LRYGB | 24 | 43.5 ± 2 | 46.1 ± 1.3 | 34.8 ± 0.8 | 12 | 1,2,3,4,5,6 |
| Chin [19] 2018 | New York | Retrospective cohort | M | GB | 37 | 16.3 ± 2 | 48.2 ± 7.9 | 40.4 ± 6.8 | 12 | 1,4,5 |
| Pellitero [20] 2012 | Spain | Prospective | M | RYGB, SG | 33 | 40.5 ± 9.9 | 50.3 ± 6.1 | 31.5 ± 4.7 | 12 | 1,2,3,6 |
| Globerman [21] 2005 | Israel | Prospective | M | VBG | 17 | 38.2 ± 2.5 | 44.3 ± 1.7 | 31.6 ± 1.5 | 11.6 ± 1.4 | 1,2,4,5 |
| Mora [22] 2013 | Spain | Prospective | M | RYGB, SG | 39 | 43.5 ± 10.5 | 46.9 ± 7.77 | 30.88 ± 5.04 | 12 | 1,2,3,4,5,6,8 |
| Bastounis [23] 1998 | Greece | Prospective | M + F | VBG | 38 (F) 19 (M) | 34.3 ± 5.9 (F) / 34.7 ± 7.7 (M) | 56.7 ± 7.7 (F) / 57.1 ± 7.4 (M) | 34.1 ± 4.8 (F) / 34.7 ± 6.5 (M) | 12 | 1,2,3,4,5,6 (F) / 1,2,3,4,5,6 (M) |
| Mingrone [24] 2002 | Italy | Prospective | M + F | BPD | 31 (F) 15 (M) | 30 - 45 | 48.3 ± 6.3 (F) / 48 ± 5.4 (M) | 35.2 ± 7.6 (F) / 30.4 ± 3.5 (M) | 12 | 6 (F) / 6 (M) |
| Alagna [25] 2006 | Italy | Prospective | M | BPD | 20 | 21 - 63 | 47.3 ± 13.1 | 33.5 ± 7 | 12 ± 1 | 1,3,4,5 |
| Woodard [26] 2012 | USA | Prospective | M | RYGB | 64 | 48.1 ± 1.3 | 48.2 ± 1.5 | 35.6 ± 1^a^ / 32.4 ± 1^b^ | 6, 12 | 1 |
| Botella-Carretero [27] 2013 | Spain | Prospective | M | BPD, RYGB, LAGB | 20 | 40 ± 10.3 | 47.05 ± 5.99 | 35 ± 6.57 | 6 | 1,2,3,4,5,6 |
| Ippersiel [28] 2013 | Belgium | Prospective | M | RYGB, SG | 21 | 40 (33 - 53) | 45.3 ± 5.6 | 31 ± 4.2 | 12 | 1,2 |
| Mihalca [29] 2014 | Romania | Prospective | M | SG | 28 | 43.07 ± 9.56 | 50.1 ± 11.19 | 35.87 ± 7.02 | 6 | 1,5,6 |
| Samavat [30] 2014 | Italy | Prospective | M | RYGB, LAGB,BPD, SG | 55 | 42.3 ± 11.6 | 46.6 ± 7.4 | 37.5 ± 6.7^a^ / 32.2 ± 6.8^b^ | 6, 12 | 1,2,3,4,5,6 |
| Legro [31] 2015 | USA | Prospective cohort | M | RYGB | 6 | 37.5 (30 - 40) | 48 ± 7 | 35 ± 7a / 32 ± 7b | 6, 12 | 1,3,6,10,11,12,14 |
| Sarwer [32] 2015 | Pennsylvania | Prospective cohort | M | RYGB | 32 | 48 (24 - 64) | 45.1 (37.3 - 64.6) | NR | 12 | 1,2,5,6,8 |
| Kun [33] 2015 | China | Retrospective cohort | M | RYGB | 39 | 45.2 ± 12.3 | 41.2 ± 8.5 | 32.1 ± 7.3 | 12 | 1 |
| Boonchaya-Anant [34] 2016 | Thailand | Prospective | M | RYGB, SG | 29 | 31 ± 8 | 56.9 ± 11.7 | 42.9 ± 9 | 6 | 1,2,3,6 |
| Gao [35] 2018 | China | Prospective | M | LSG | 30 | 33 ± 9.5 | 40.2 ± 5.2 | 30.8 ± 4.4 | 6 | 1,2,3,4,5,6 |
| Liu [36] 2018 | China | Retrospective | M | RYGB | 45 | 47 ± 9.97a / 46.5 ± 9.71b | 32.81 ± 4.04 | 25.48 ± 3.29^a^ / 25.41 ± 3.36^b^ | 6, 12 | 1,2 |
| Samavat [37] 2018 | Italy | Prospective | M | LRYGB | 23 | 38 ± 9 | 45.8 ± 7.4 | 34.7 ± 5.3 | 6 | 1,2,3,4,5,6,10,11,12,13,14 |
| Fariello [38] 2021 | Brazil | Prospective | M | RYGB | 15 | 20 - 50 | 45.7 ± 8.3 | 36.1 ± 6.4^a^ / 28.0 ± 2.8^b^ | 6, 12 | 1,2,3,4,5,6,10,11,12,13,14 |
| Oncel [39] 2021 | Turkey | Prospective | M | LSG | 40 | 35.70 ± 4.22 | 47.20 ± 6.62 | 35.89 ± 4.95 | 6 | 1 |
| Zhu [40] 2019 | China | Prospective | M | LSG | 56 | 30.8 ± 7.8 | 41.9 ± 5.8 | 26.1 ± 4.3 | 12 | 1,2 |
| Ernst [41] 2013 | Switzerland | Prospective | F | RYGB | 36 | 41.2 ± 1.6 | 44.5 ± 0.8 | 27.9 ± 0.6 | 12 | 1,2,6 |
| Legro [42] 2012 | USA | Prospective cohort | F | RYGB | 29 | 34.5 ± 4.3 | 49 ± 7 | NR | 6, 12 | 7 |
| Sarwer [43] 2014 | Pennsylvania | Prospective cohort | F | RYGB, LAGB | 106 | 41 (34 - 48) | 44.5 (41.4 - 49.7) | NR | 12 | 1,3,4,5,6,7 |
| Kjaer [44] 2017 | Denmark | Prospective cohort | F | RYGB | 31 | 34 (22 - 49) | 44.1 ± 5.8 | 32.4 ± 9.8^a^ / 30.3 ± 5.8^b^ | 6, 12 | 1,2,3,4,5,6 |
| Eid [45] 2014 | Pennsylvania | Prospective | F | RYGB | 14 | 36.3 ± 8.4 | 44.8 ± 1.6 | 32.4 ± 0^a^ / 29.2 ± 5.9^b^ | 6, 12 | 1,2,4,5,9 |
| Escobar-Morreal [46] 2005 | Spain | Prospective | F | BPD, LGB | 17 | 29.8 ± 5.3 | 50.7 ± 7.1 | NR | 12 ± 5 | 1,2 |
| Bhandari [47] 2016 | India | Prospective | F | SG | 75 | 28 ± 5 | 43.77 ± 5.9 | 31.71 ± 3.2 | 6 | 9 |
| Turkmeen [48] 2015 | Sweden | NR | F | LRYGB | 8 | 31.4 ± 7.41 | 47.2 ± 8.85 | 35.7 ± 8.01^a^ / 32.82 ± 9.3^b^ | 6, 12 | 1,6,9 |
| Dixon [49] 2011 | Australia | NR | F | Lap-Band | 42 | 34.0 ± 6.5 | 45.3 ± 7.3 | 36.4 ± 6.8 | 12 | 1,6 |
| Carette [50] 2011 | France | Prospective cohort | M | GB, SG | 46 | 38.9 ± 7.9 | 44.1 ± 5.7 | 33.2 ± 5.4^a^ / 31.4 ± 5.3^b^ | 6, 12 | 12,13,14 |
| Bond [51] 2011 | USA | Prospective | F | RYGB, LAGB | 54 | 43.3 ± 9.5 | 45.1 ± 6.8 | NR | 6 | 7 |
| Whitcomb [52] 2012 | USA | Prospective cohort | F | LGB, LSG | 98 | 43.3 ± 11.8 | 39.7 ± 6.2 | 34.4a ± 5.4^a^ / 34.0 ± 5.6^b^ | 6, 12 | 7 |
| Hernández [53] 2013 | Spain | Prospective | F | LBPD | 80 | 43.5 ± 9.2 | 52.2 ± 8.2 | NR | 6, 12 | 7 |
| Goitein [54] 2015 | Israel | Prospective | F | LRYGB, SG | 34 | 38.4 ± 9.1 | 44.4 ± 5.5 | 32.5 ± 5.1 | 6 | 7 |
| Pichlerova [55] 2019 | Czech Republic | Prospective | F | LAGB, BPD,Gastric Plication | 60 | 41.7 ± 10.8 | 43.7 ± 5.99 | 36.4^a^ | 6, 12 | 7 |
| Cherick [56] 2019 | France | Prospective | F | SG, RYGB | 36 | 37 ± 13 | 41 ± 7 | 29 ± 5 | 6 | 7 |
| Lechmiannandan [57] 2019 | Malaysia | Prospective | F | SG, GB | 52 | 38.77 ± 6.7 | 39.89 ± 6.9 | 30.32 ± 5.4 | 6 | 7 |
| Assimakopoulos [58] 2011 | Greece | Prospective | F | BPD-LL, SG, / RYGB-LL | 59 | 18 - 56 | 51.9 ± 9.92 | 31.8 ± 4.92 | 12 | 7 |
| Efthymiou [59] / 2015 | Greece | Prospective | M + F | SG, RYGB, BPD | 50 | 37.3 ± 9.6 (M) / 37.2 ± 10.7 (F) | 50.66 ± 7.9 | NR | 6, 12 | 7,8 |
| Akan [60] 2018 | Turkey | Prospective | F | LSG | 53 | 34.85 ± 9.38 | 47.43 ± 6.37 | 37.77 ± 5.2 | 12 | 7 |

R = not reported, BMI = body mass index, F = Females, M = Males, n = number of participants

RYGB = Roux-en-Y gastric bypass, LRYGB = laparoscopic Roux-en-Y gastric bypass, SG = sleeve gastrectomy, LSG = laparoscopic sleeve gastrectomy, LAGB = laparoscopic adjustable gastric band, LGB = laparoscopic gastric banding, BPD = biliopancreatic diversion, LBPD = laparoscopic biliopancreatic diversion, RYGB-LL = Roux-en-Y gastric bypass with long limb, BPD-LL = biliopancreatic diversion with Roux-en-Y reconstruction, GB = gastric bypass, VBG = Vertical banded gastroplasty, Lap-Band = laparoscopic banding
BMI = body mass index, FSFI = Female Sexual Function Index, IIEF- erectile function = International Index of Erectile Function - component of IIEF, TT = total testosterone, FT = free testosterone, LH = luteinizing hormone, FSH = follicle stimulating hormone, SHBG = sex hormone–binding globulin, E2 = estradiol.

Outcome Indicators: 1 = (TT), 2 = (FT), 3 = (E2), 4 = (FSH), 5 = (LH), 6 = (SHBG), 7 = (FSFI), 8 = (IIEF- erectile function), 9 = Menstrual dysfunction, 10 = sperm concentration, 11 = sperm morphology, 12 = sperm motility, 13 = sperm count, 14 = semen volume, ^a^ = 6 months follow-up ^b^ = 12 months follow-up

| Table s3. Risk of bias assessment: MINORS criterion | | | | | | | | | | | |
| --- | --- | --- | --- | --- | --- | --- | --- | --- | --- | --- | --- |
| **Study** | **A stated objective of the study was to provide a theoretical basis for the study.** | **Inclusion of consecutive patients** | | **Prospective collection of data** | **Endpoints appropriate to the study aim** | | **Unbiased assessment of the study endpoint** | **Follow-up period appropriate to the aim of the study** | **Loss to follow-up not exceeding 5%** | **Prospective calculation of the study size** | **Total** |
| Chin 2018 | 2 | | 2 | 2 | 2 | 2 | | 2 | 2 | 0 | 14 |
| Fariello 2021 | 2 | | 2 | 2 | 2 | 2 | | 2 | 2 | 2 | 16 |
| Oncel 2021 | 2 | | 2 | 2 | 2 | 2 | | 1 | 2 | 0 | 13 |
| Zhu 2019 | 2 | | 2 | 2 | 2 | 2 | | 1 | 2 | 0 | 13 |
| Ernst, 2013 | 2 | | 1 | 2 | 2 | 2 | | 2 | 2 | 0 | 13 |
| Sarwer 2014 | 2 | | 1 | 1 | 2 | 2 | | 2 | 1 | 2 | 13 |
| Bastounis 1998 | 1 | | 1 | 2 | 2 | 2 | | 2 | 2 | 0 | 12 |
| Globerman, 2005 | 2 | | 2 | 2 | 2 | 2 | | 2 | 1 | 0 | 13 |
| Mingrone 2002 | 2 | | 0 | 2 | 2 | 2 | | 2 | 1 | 0 | 11 |
| Alagna 2006 | 2 | | 1 | 2 | 2 | 2 | | 2 | 1 | 0 | 12 |
| Pellitero 2012 | 2 | | 0 | 2 | 2 | 2 | | 2 | 1 | 0 | 11 |
| Woodard 2012 | 2 | | 2 | 2 | 2 | 2 | | 2 | 1 | 0 | 13 |
| Botella-Carretero 2013 | 2 | | 2 | 2 | 2 | 2 | | 1 | 2 | 2 | 15 |
| Ippersiel 2013 | 2 | | 2 | 2 | 2 | 2 | | 2 | 0 | 0 | 12 |
| Mora 2013 | 2 | | 2 | 2 | 2 | 2 | | 2 | 0 | 0 | 12 |
| Aarts 2014 | 2 | | 1 | 2 | 2 | 2 | | 2 | 2 | 0 | 13 |
| Mihalca 2014 | 2 | | 2 | 2 | 2 | 2 | | 1 | 0 | 0 | 11 |
| Samavat 2014 | 2 | | 2 | 2 | 2 | 2 | | 2 | 1 | 0 | 13 |
| Legro 2015 | 2 | | 1 | 2 | 2 | 2 | | 2 | 0 | 2 | 13 |
| Sarwer 2015 | 2 | | 1 | 2 | 2 | 2 | | 2 | 2 | 0 | 13 |
| Kun 2015 | 2 | | 2 | 1 | 2 | 2 | | 2 | 2 | 0 | 13 |
| BoonchayaAnant 2016 | 2 | | 1 | 2 | 2 | 2 | | 1 | 1 | 0 | 11 |
| Gao 2018 | 2 | | 2 | 2 | 2 | 2 | | 1 | 1 | 0 | 12 |
| Liu 2018 | 2 | | 2 | 0 | 2 | 2 | | 2 | 1 | 0 | 11 |
| Samavat 2018 | 2 | | 2 | 2 | 2 | 2 | | 1 | 2 | 0 | 13 |
| Legro 2012 | 2 | | 2 | 2 | 2 | 2 | | 2 | 1 | 0 | 13 |
| Bond, 2011 | 2 | | 2 | 2 | 2 | 2 | | 1 | 2 | 0 | 13 |
| Whitcomb  2012 | 2 | | 1 | 2 | 2 | 2 | | 2 | 2 | 0 | 13 |
| Hernández 2013 | 2 | | 2 | 2 | 2 | 2 | | 2 | 2 | 0 | 14 |
| Goitein 2015 | 2 | | 1 | 2 | 2 | 2 | | 1 | 1 | 0 | 11 |
| Pichlerova 2019 | 2 | | 2 | 2 | 2 | 2 | | 2 | 0 | 0 | 12 |
| Assimakopoulos 2011 | 2 | | 2 | 2 | 2 | 2 | | 2 | 2 | 0 | 14 |
| Cherick 2019 | 2 | | 2 | 2 | 2 | 2 | | 1 | 1 | 0 | 12 |
| Lechmiannandan 2019 | 2 | | 1 | 2 | 2 | 2 | | 1 | 2 | 0 | 12 |
| Efthymiou 2015 | 2 | | 2 | 2 | 2 | 2 | | 2 | 2 | 0 | 14 |
| Akan 2018 | 2 | | 2 | 2 | 2 | 2 | | 2 | 2 | 0 | 14 |
| Turkmen 2014 | 2 | | 2 | 2 | 2 | 2 | | 2 | 2 | 0 | 14 |
| Dixon, 2002 | 2 | | 2 | 2 | 2 | 2 | | 2 | 2 | 2 | 16 |
| Bhandari 2016 | 2 | | 2 | 2 | 2 | 2 | | 1 | 2 | 0 | 13 |
| Carette 2019 | 2 | | 1 | 2 | 2 | 2 | | 2 | 2 | 2 | 15 |
| Eid 2014 | 2 | | 2 | 2 | 2 | 2 | | 2 | 2 | 0 | 14 |
| Kaejar 2017 | 2 | | 1 | 2 | 2 | 2 | | 2 | 1 | 0 | 12 |
| Escobar 2005 | 2 | | 1 | 2 | 2 | 2 | | 1 | 2 | 2 | 14 |

|  |  |  |  |  |  |  |  |  |  |  |  |  |  |  |  |  |  |  |
| --- | --- | --- | --- | --- | --- | --- | --- | --- | --- | --- | --- | --- | --- | --- | --- | --- | --- | --- |

**Figure S1.** PRISMA flow diagram


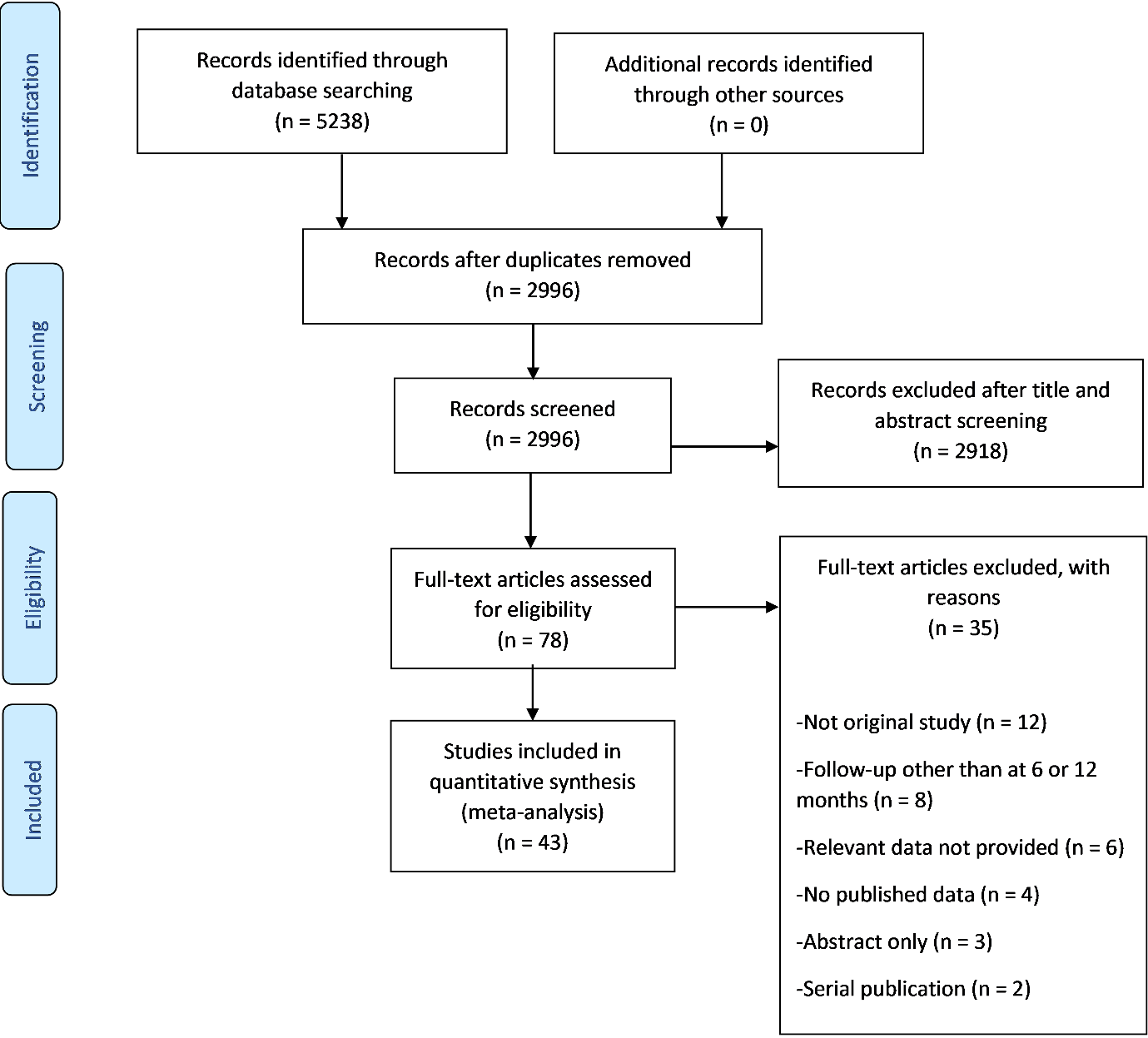


**Figure S2.** Forest plot for erectile function component of IIEF at 12-month follow-up


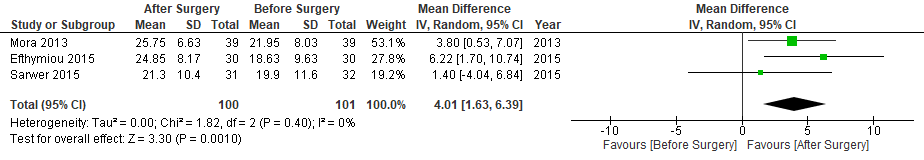


**Figure S3.** Forest plots for male sex hormones at 12-month follow-up (a) male TT (nM), (b) male FT (nM), (c) male estradiol (pg/mL), (d) male LH (mIU/mL), (e) male FSH (mIU/mL), (f) male SHBG (nM)

**a**

**
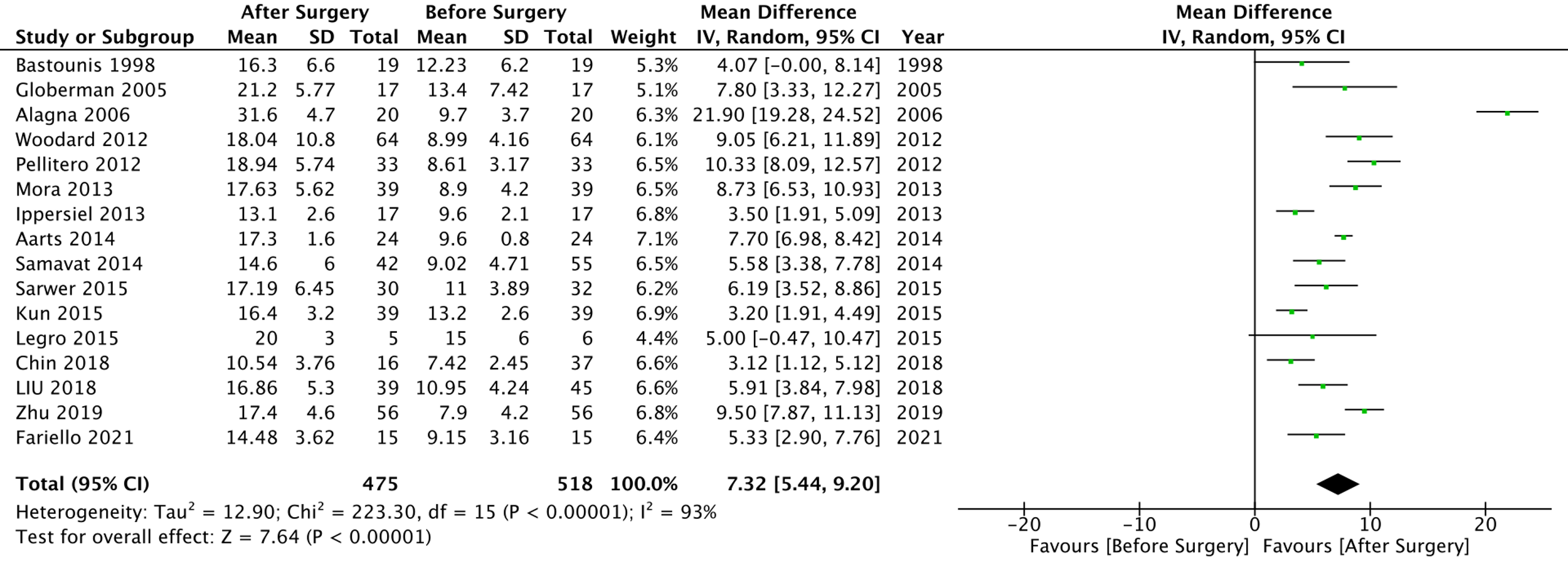
**

**b**

**
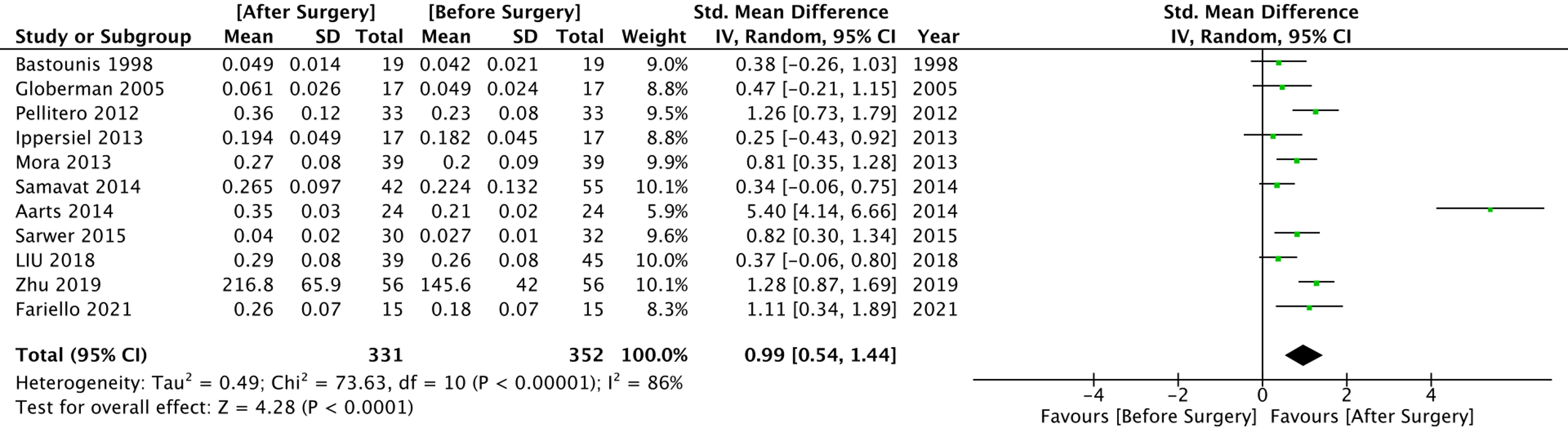
**

**c**

**
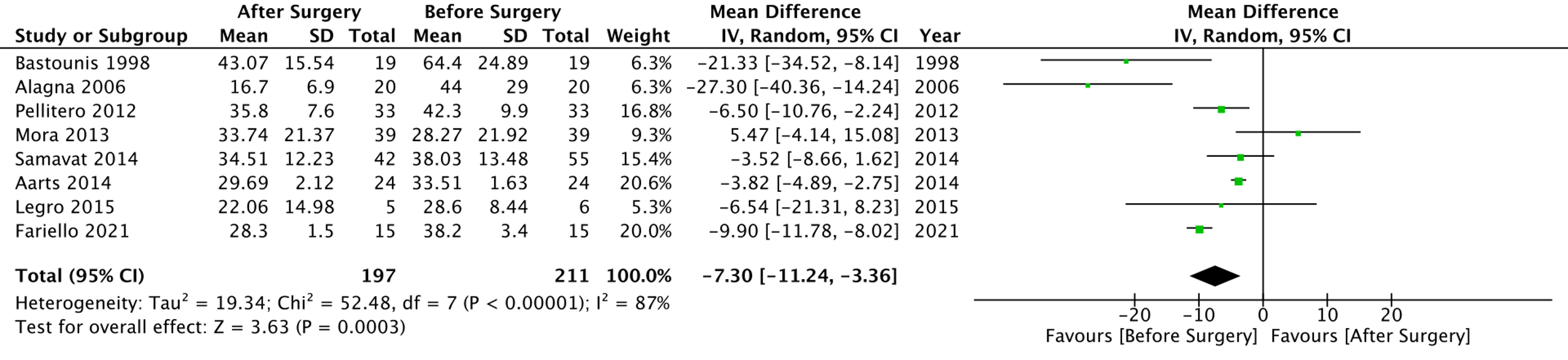
**

**d**

**
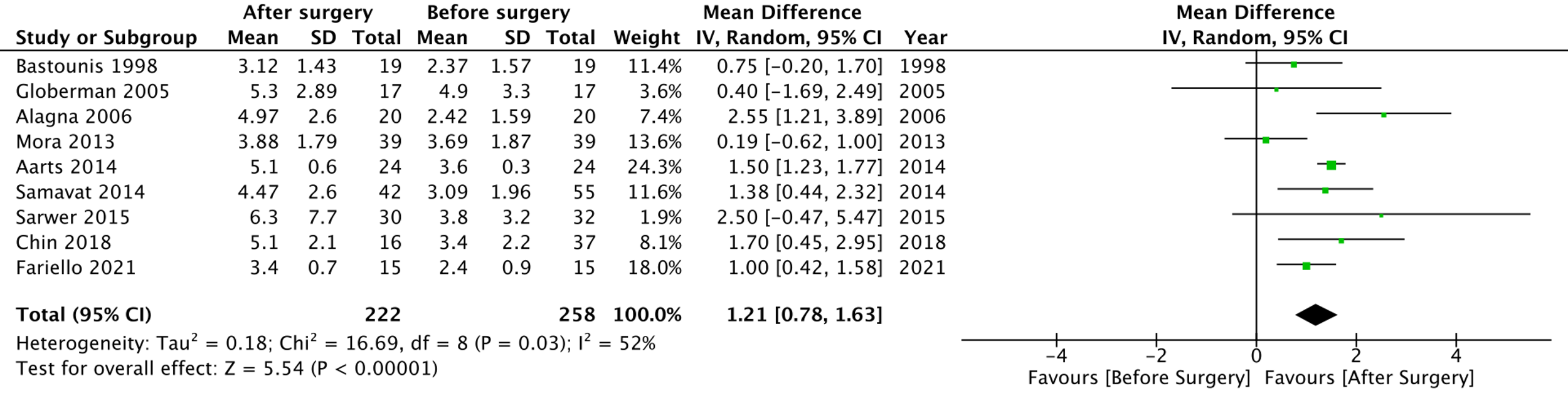
**

**e**

**
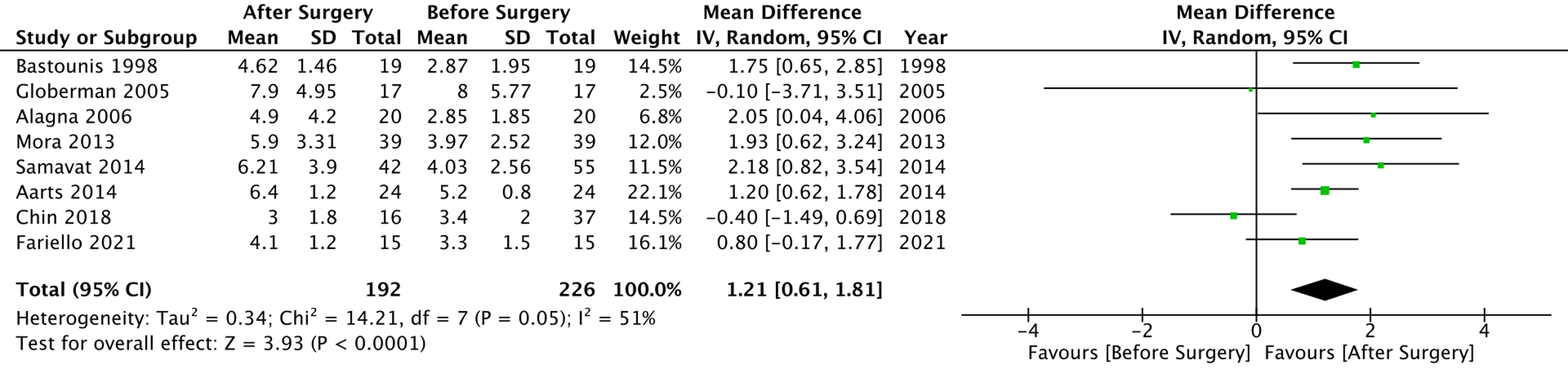
**

**f**

**
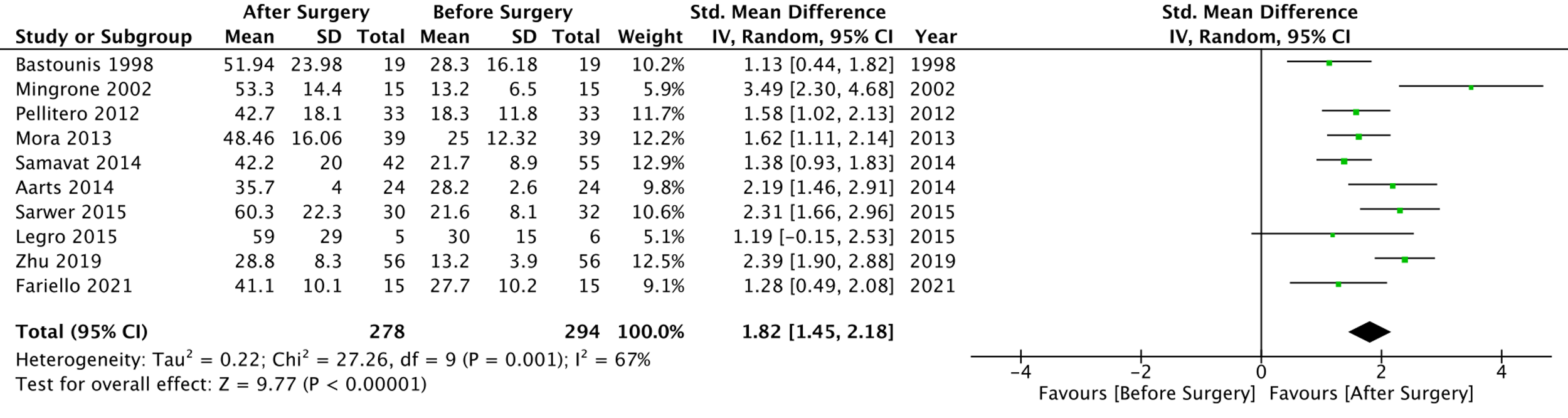
**

**Figure S4.** Forest plots for semen analysis at 12 months of follow-up (a) total sperm count (10^6^), (b) semen volume (mL), (c) sperm motility (%), (d) sperm morphology (%), (e) sperm concentration (million / mL)

**a**


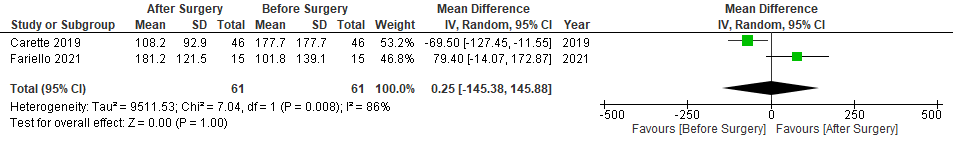


**b**


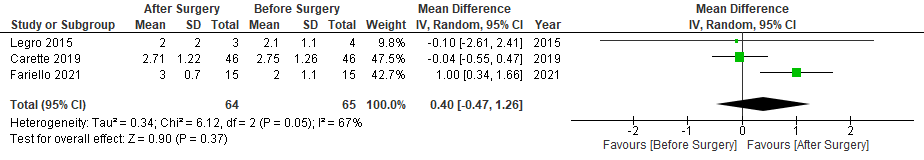


**c**


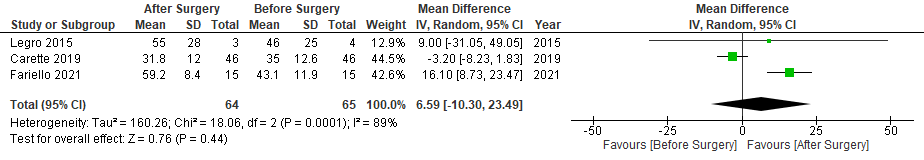


**d**


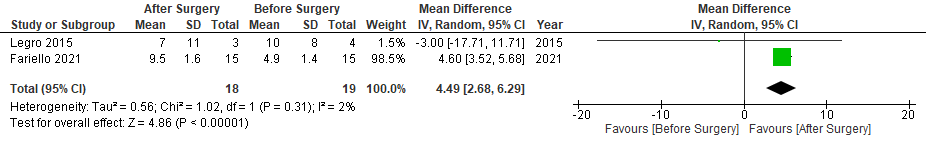


**e**


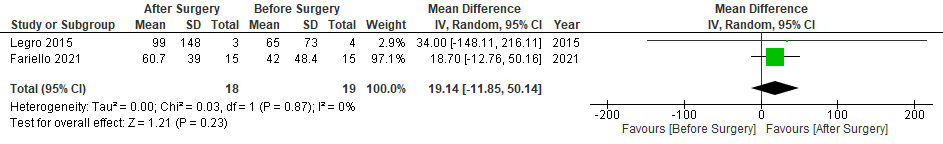


**Figure S5.** Forest plot of the total FSFI score at 12 months of follow-up


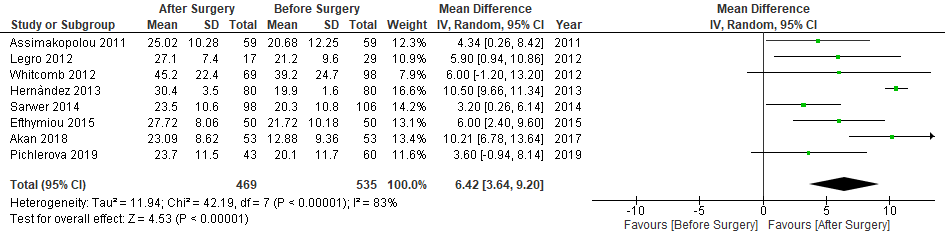


**Figure S6.** Forest plots for female sex hormones at 12-month follow-up (a) female TT (nM), (b) female FT (nM), (c) female estradiol (pg/mL), (d) female LH (mIU/mL), (e) female FSH (mIU/mL), (f) female SHBG (nM)

**a**

**
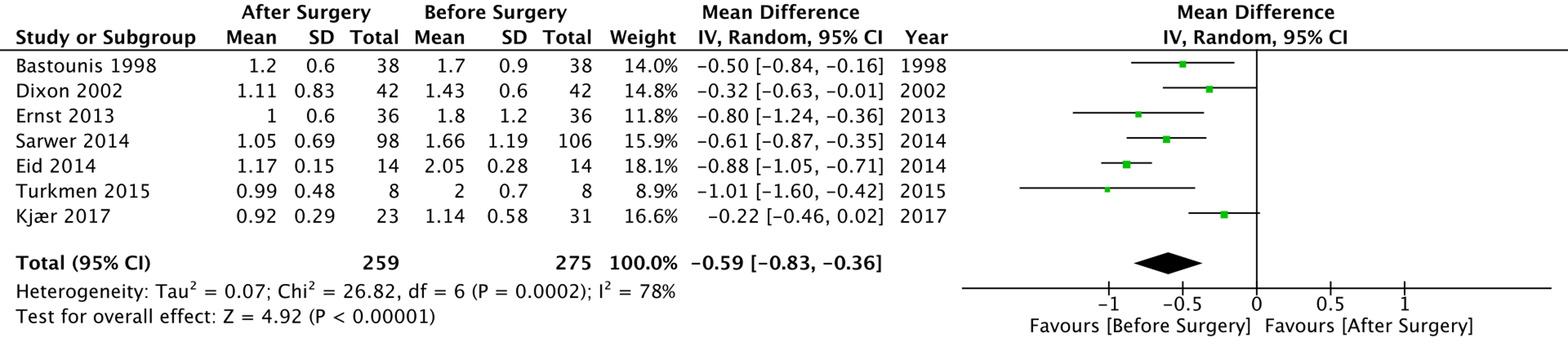
**

**b**

**
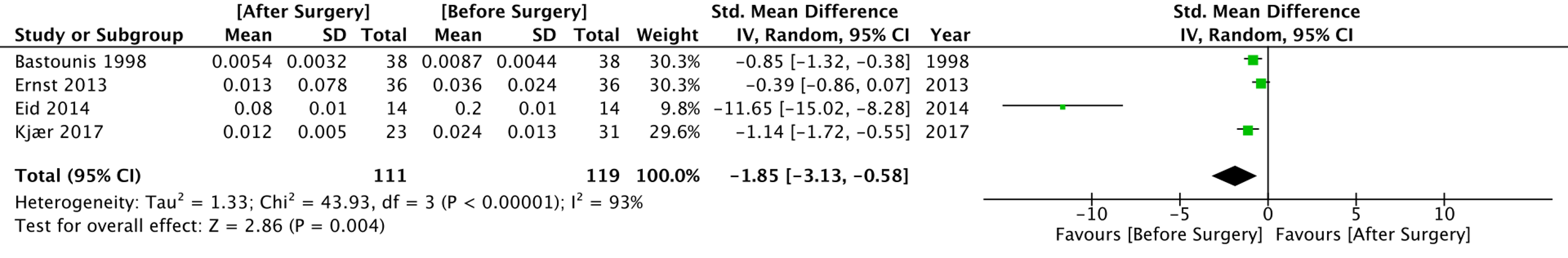
**

**c**

**
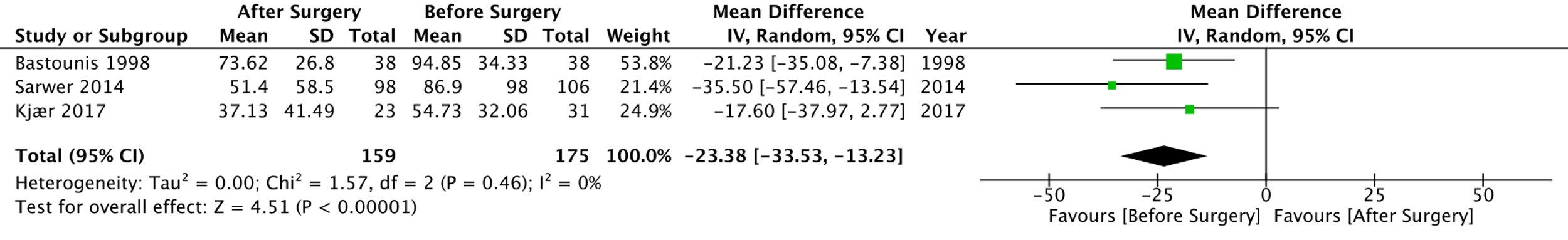
**

**d**

**
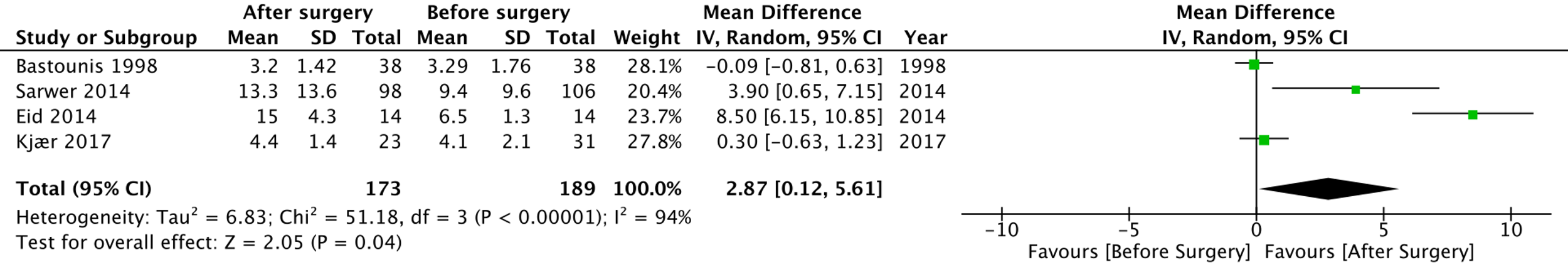
**

**e**

**
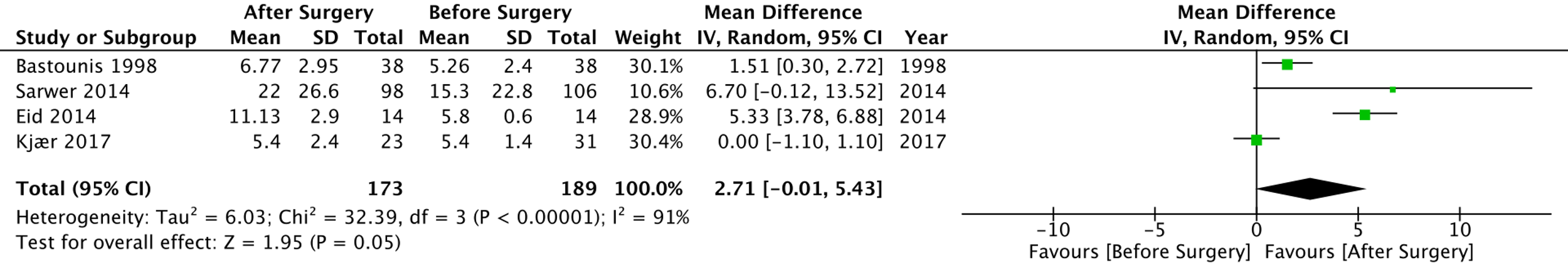
**

**f**

**
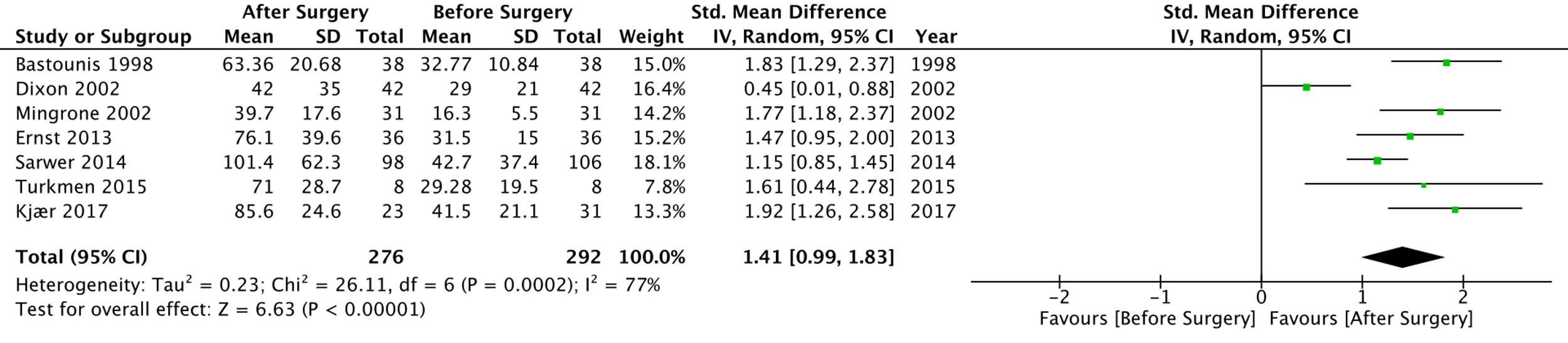
**

**Figure S7.** Forest plot for menstrual irregularity at 12-month follow-up


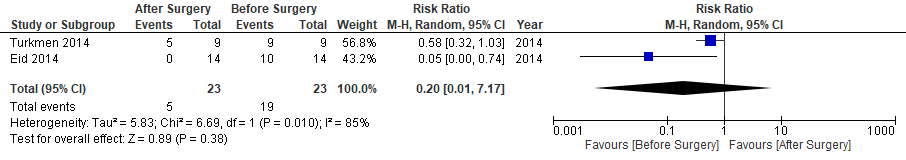


**Figure S8.** Forest plots for male sex hormones at 6-month follow-up (a) male TT (nM), (b) male FT (nM), (c) male estradiol (pg/mL), (d) male LH (mIU/mL), (e) male FSH (mIU/mL), (f) male SHBG (nM)

**a**

**
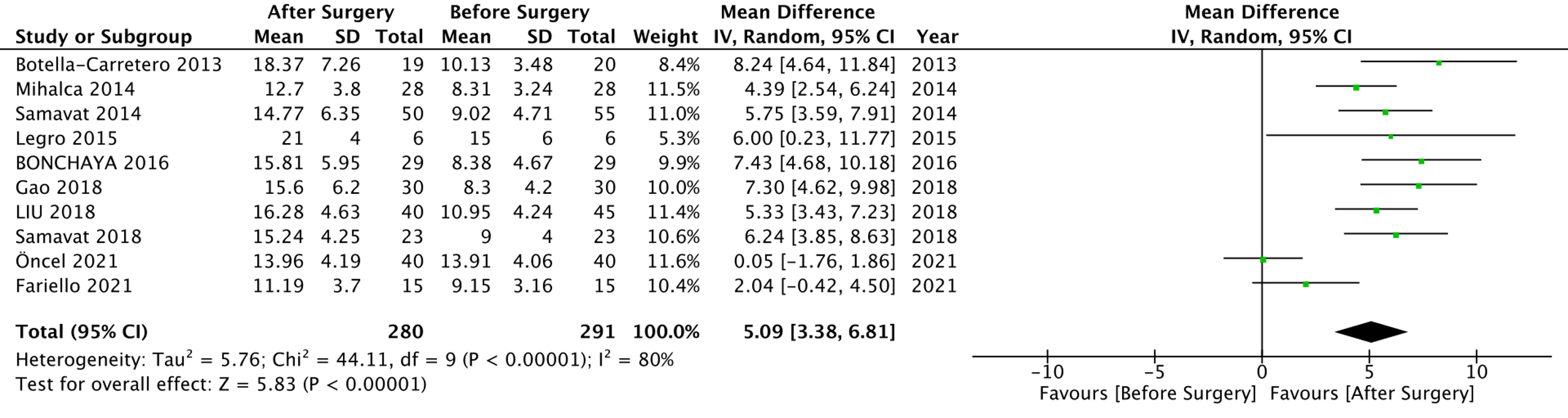
**

**b**

**
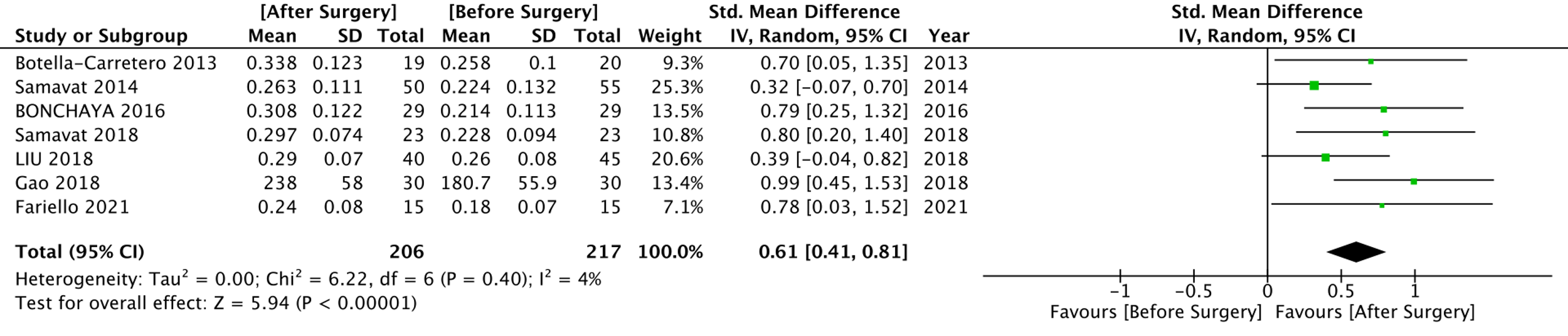
**

**c**

**
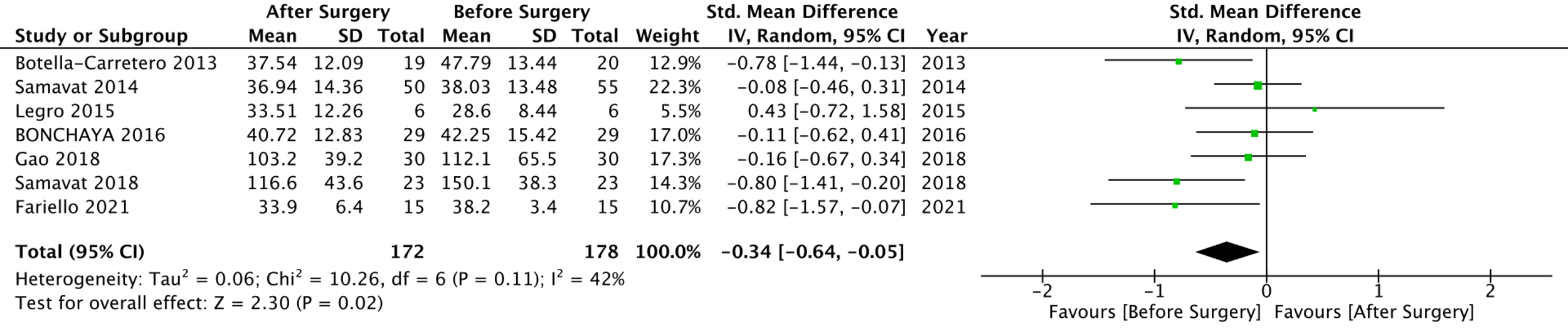
**

**d**

**
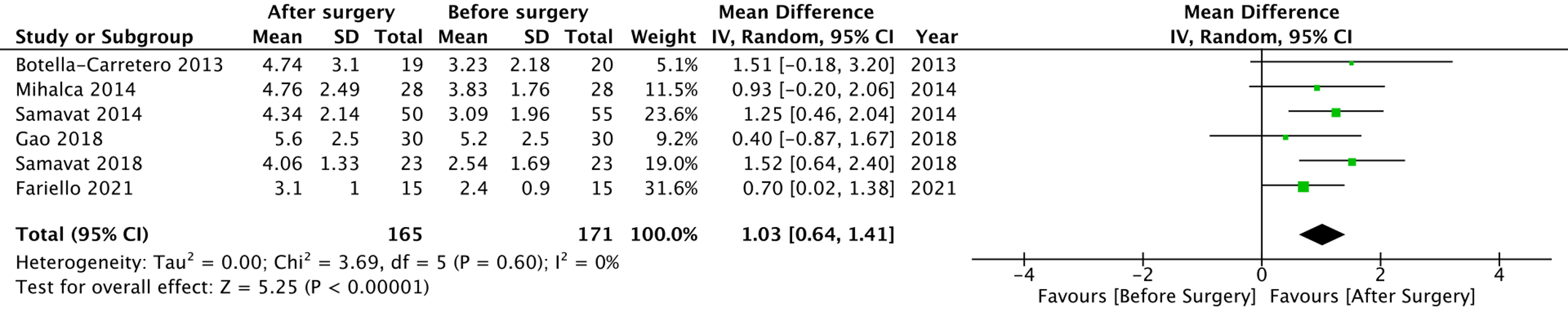
**

**e**

**
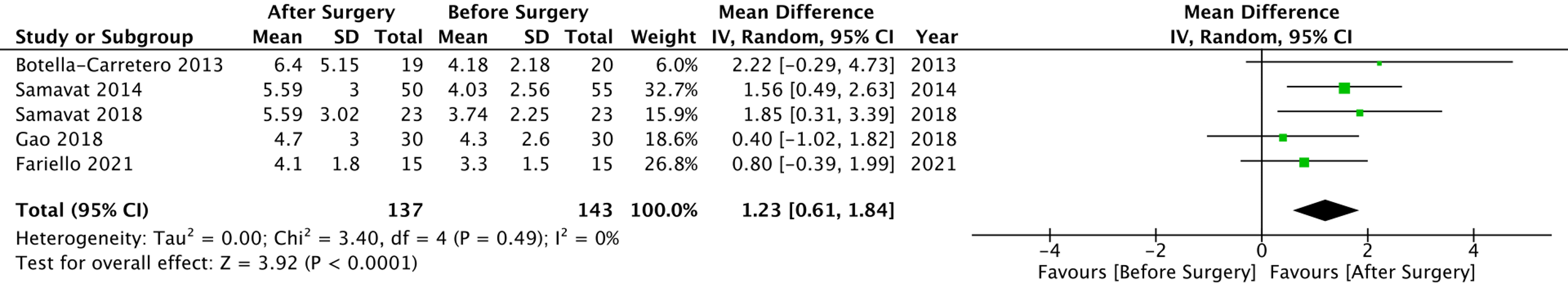
**

**f**

**
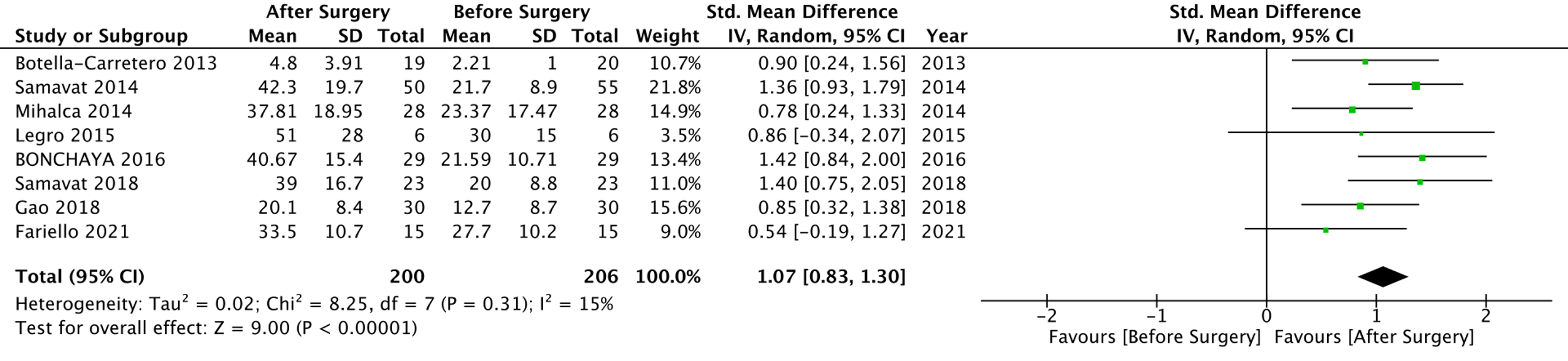
**

**Figure S9.** Forest plots for semen analysis at 6 months of follow-up (a) total sperm count (10^6^), (b) semen volume (mL), (c) sperm motility (%), (d) sperm morphology (%), (e) sperm concentration (million/mL)

**a**

**b**
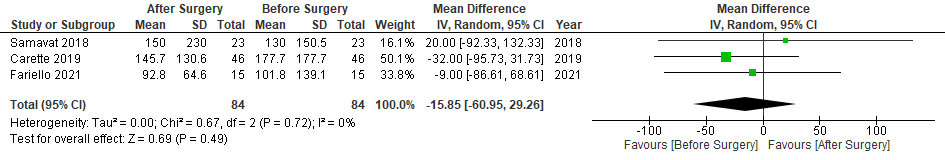


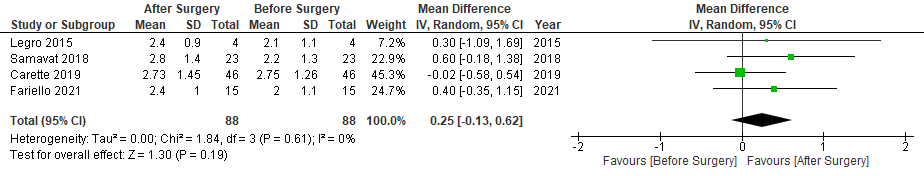


**c**


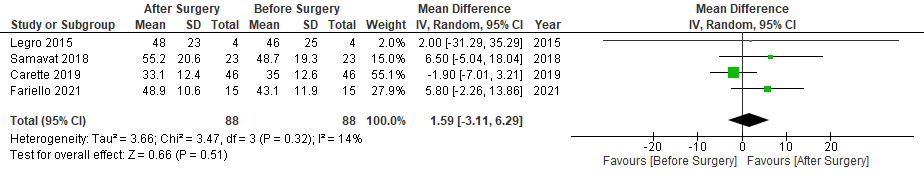


d


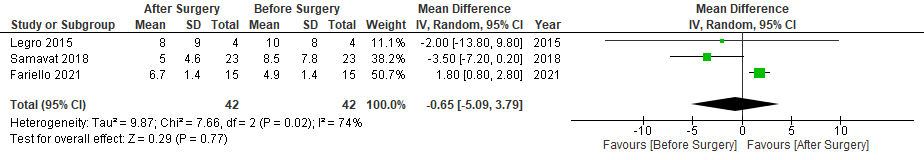


**e**


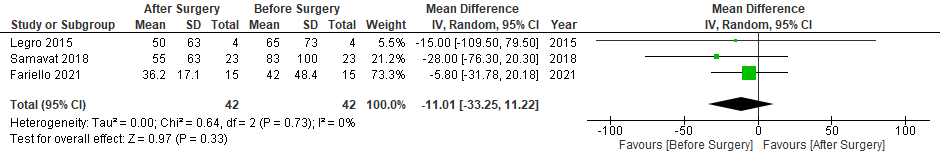


**Figure S10.** Forest plot for the total FSFI score at 6 months of follow-up


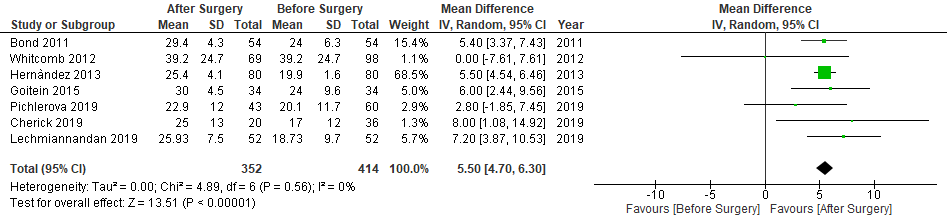


**Figure S11.** Forest plots for female sex hormones at 6-month follow-up (a) female TT (nM), (b) female FT (nM), (c) female LH (mIU/mL), (d) female FSH (mIU/mL), (e) female SHBG (nM)

**a**

**
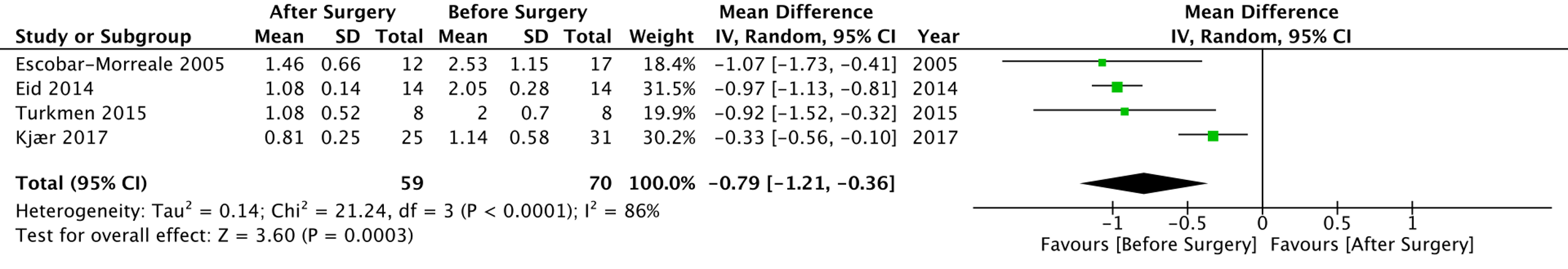
**

**b**

**
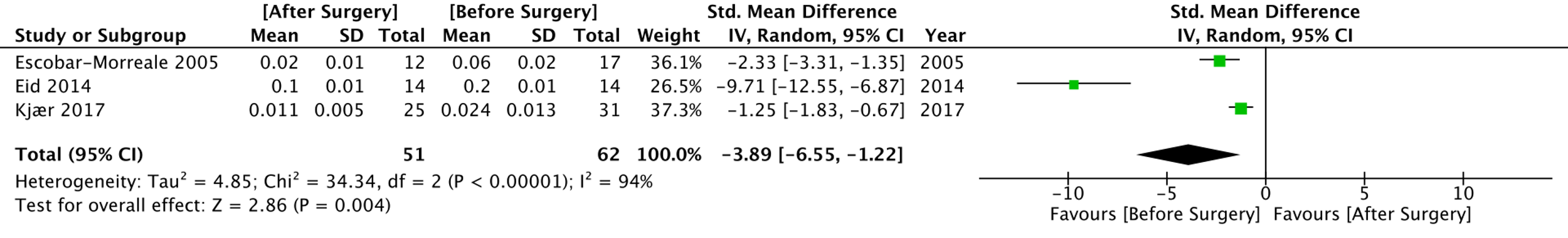
**

**c**

**
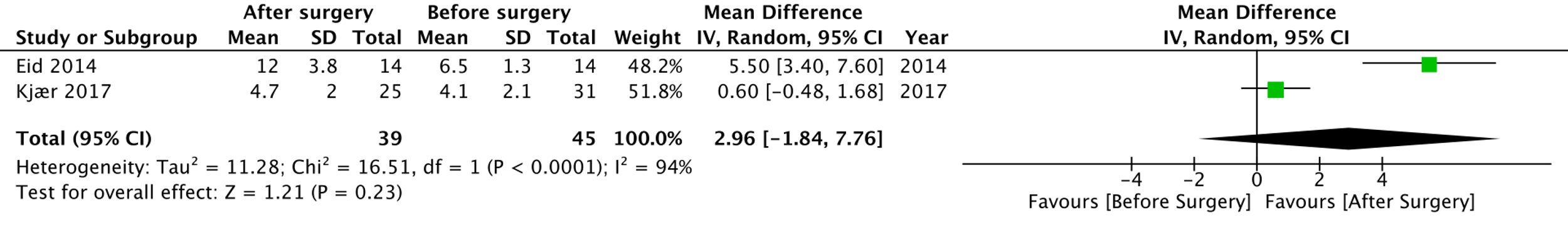
**

**d**

**
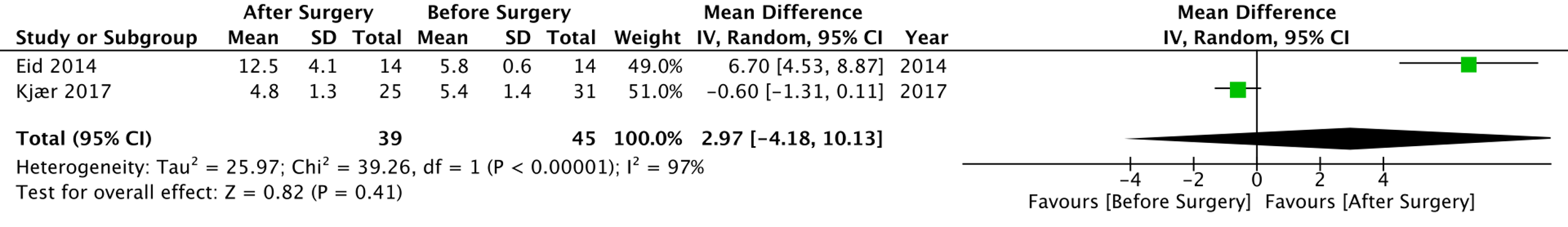
**

**e**

**
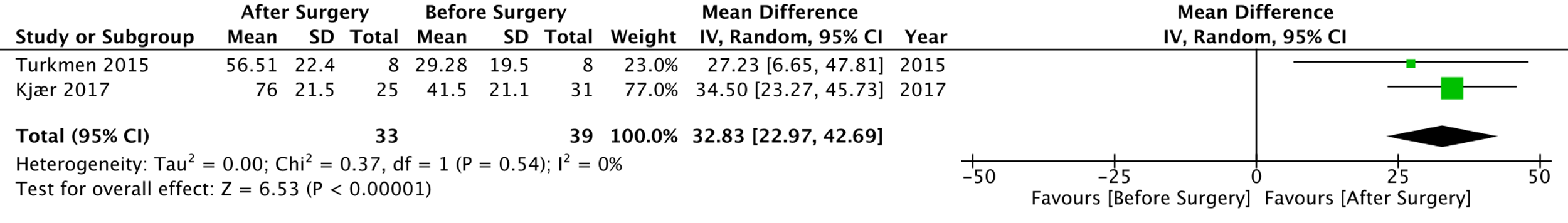
**

**Figure S12.** Forest plot of menstrual irregularity at 6 months of follow-up


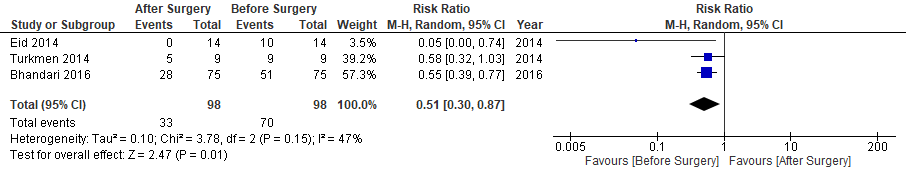


**Figure S13.** Funnel plots for male sex hormones at 12-month follow-up (a) TT, (b) FT, (c) SHB

**
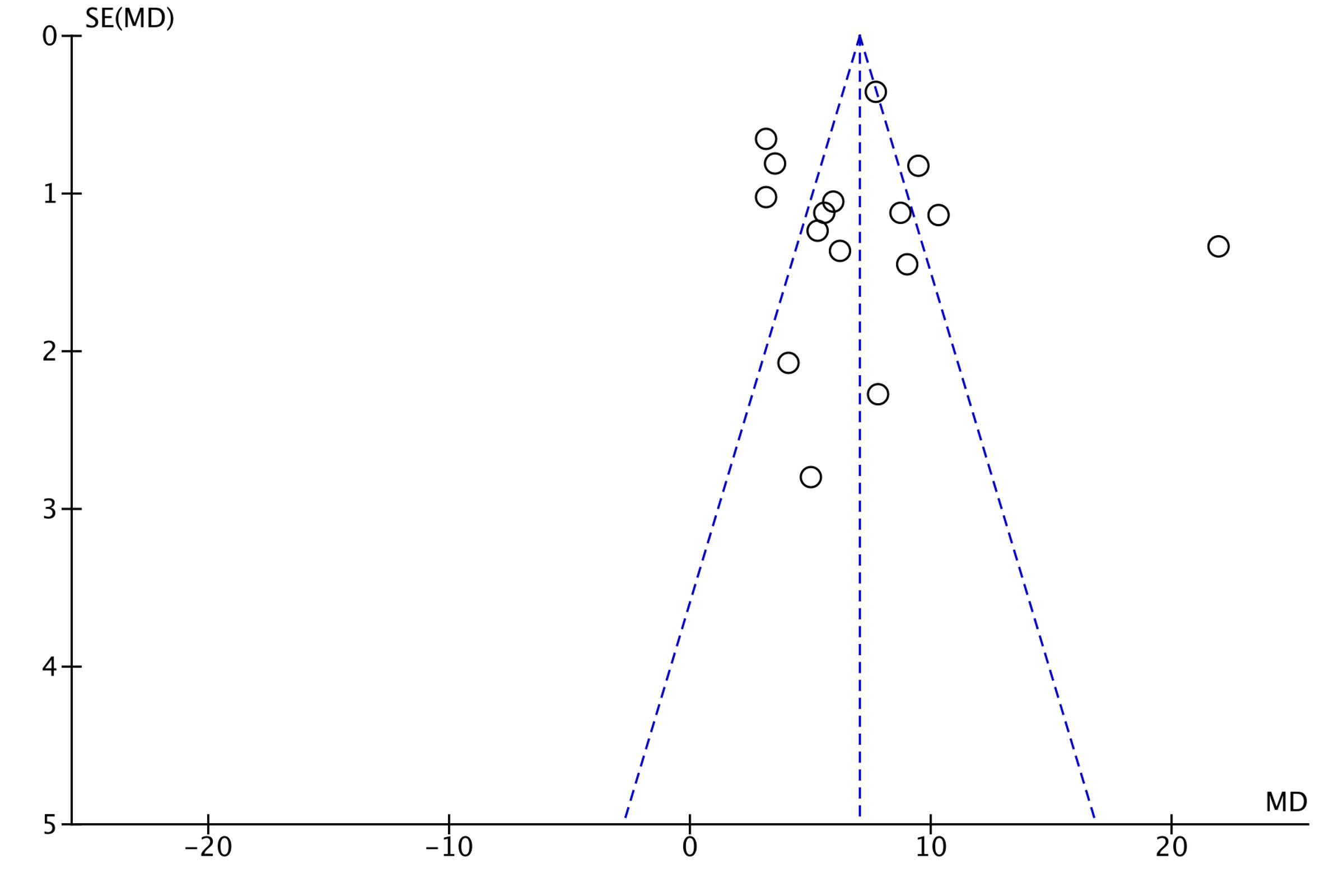
**

**a**

**b**

**
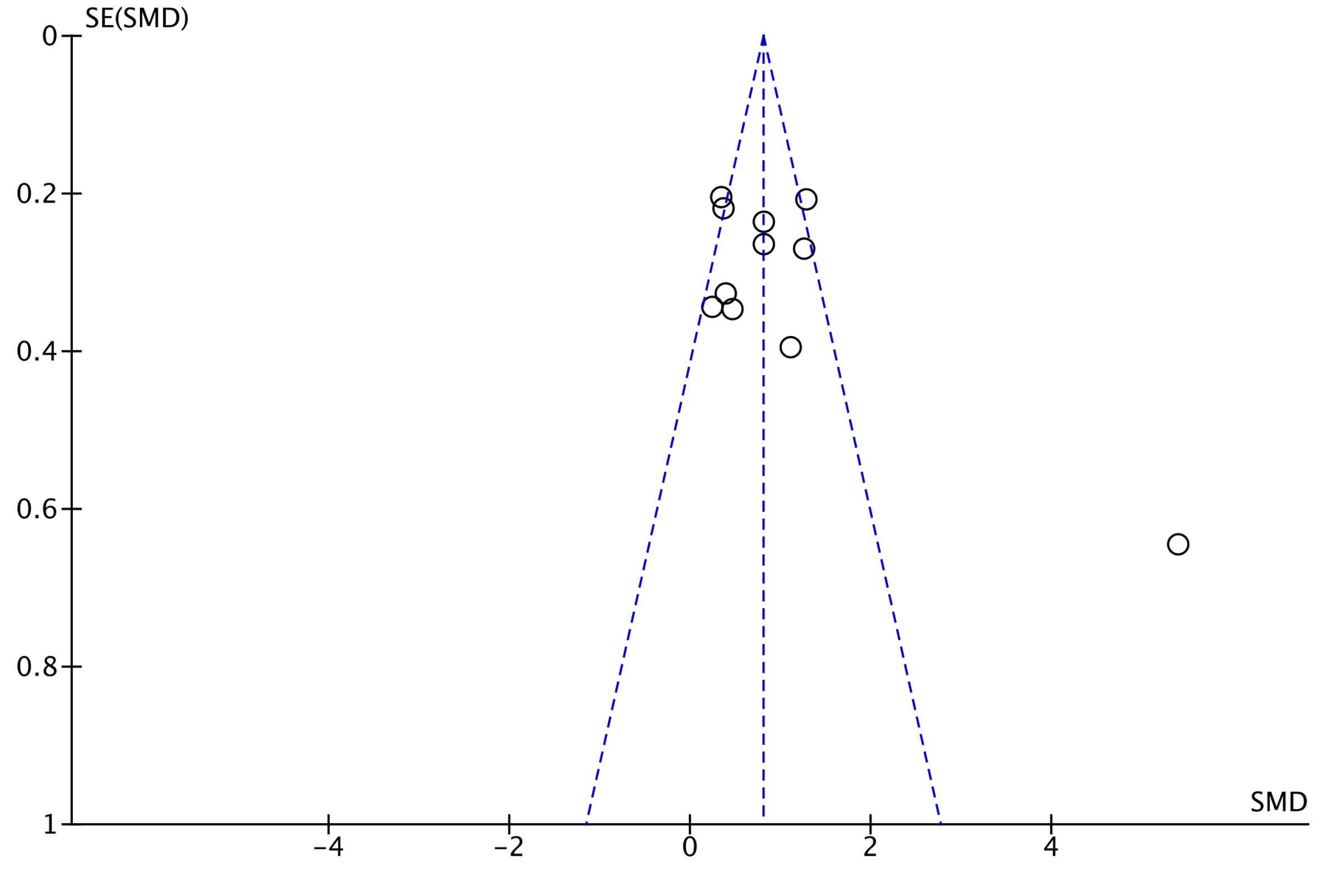
**

**
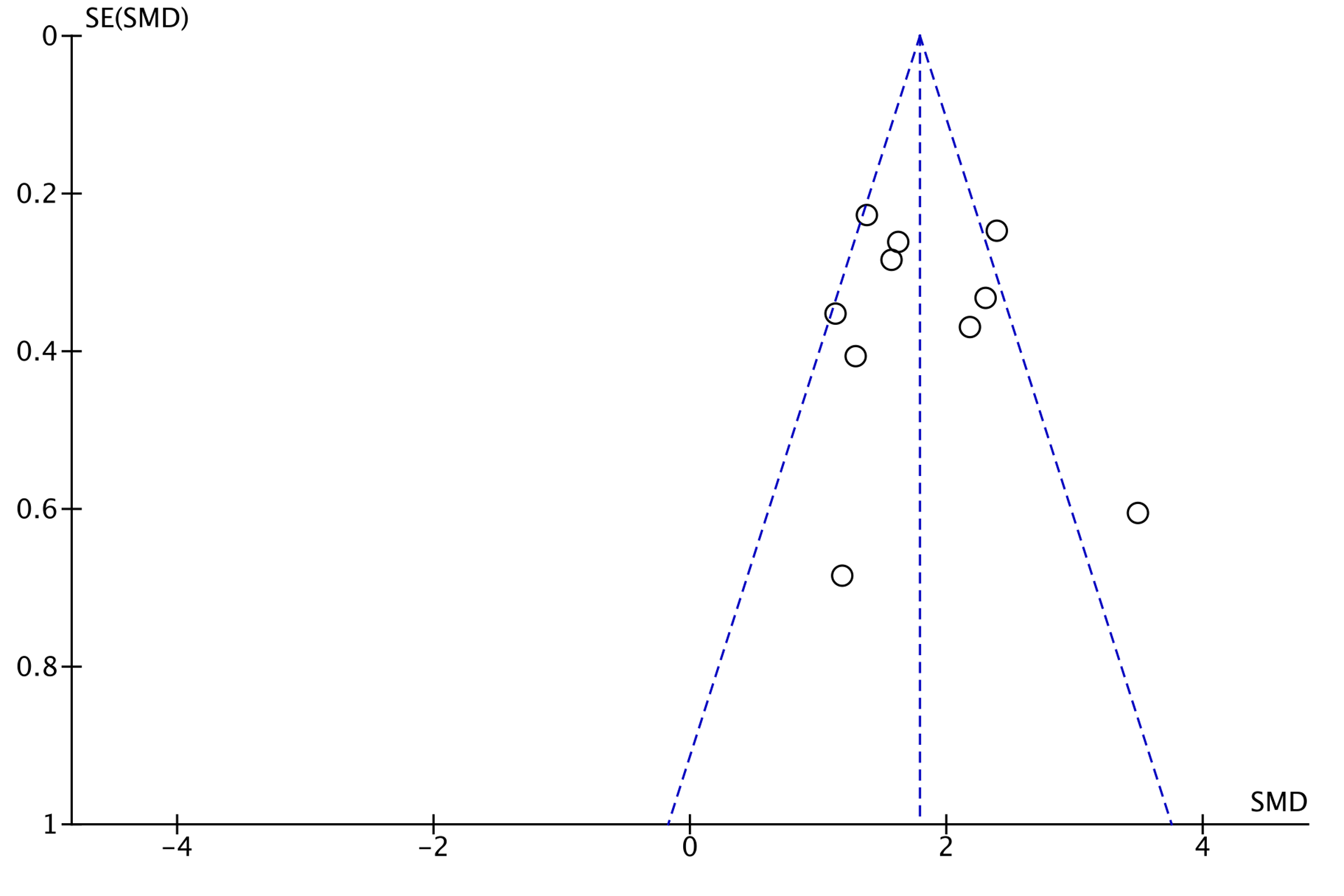
**

**c**

**Additional educational resources:**

1. Chavez‐Tapia NC, Tellez‐Avila FI, Barrientos‐Gutierrez T, Mendez‐Sanchez N, Lizardi‐Cervera J, Uribe M. Bariatric surgery for non‐alcoholic steatohepatitis in obese patients. Cochrane Database of Systematic Reviews 2010, Issue 1. Art. No.: CD007340. DOI: 10.1002/14651858.CD007340.pub2. Accessed 01 February 2022.
2. Jefferys AE, Siassakos D, Draycott T, Akande VA, Fox R. Deflation of gastric band balloon in pregnancy for improving outcomes. Cochrane Database of Systematic Reviews 2013, Issue 4. Art. No.: CD010048. DOI: 10.1002/14651858.CD010048.pub2. Accessed 01 February 2022.
3. Opray N, Grivell RM, Deussen AR, Dodd JM. Directed preconception health programs and interventions for improving pregnancy outcomes for women who are overweight or obese. Cochrane Database of Systematic Reviews 2015, Issue 7. Art. No.: CD010932. DOI: 10.1002/14651858.CD010932.pub2. Accessed 01 February 2022.
4. Colquitt JL, Pickett K, Loveman E, Frampton GK. Surgery for weight loss in adults. Cochrane Database of Systematic Reviews 2014, Issue 8. Art. No.: CD003641. DOI: 10.1002/14651858.CD003641.pub4. Accessed 01 February 2022.
